# Identification, evolution, and expression of GDSL-type Esterase/Lipase (GELP) gene family in three cotton species: a bioinformatic analysis

**DOI:** 10.1186/s12864-023-09717-3

**Published:** 2023-12-21

**Authors:** Lisheng Duan, Fei Wang, Haitao Shen, Shuangquan Xie, Xifeng Chen, Quanliang Xie, Rong Li, Aiping Cao, Hongbin Li

**Affiliations:** Key Laboratory of Xinjiang Phytomedicine Resource and Utilization of Ministry of Education, Key Laboratory of Oasis Town and Mountain-Basin System Ecology of Xinjiang Production and Construction Corps, College of Life Sciences, Shihezi University, Shihezi, 832003 China

**Keywords:** Cotton GDSL esterase/lipase, Whole genome duplication, Evolution, Fiber growth, Tissue development, Biotic and abiotic stress, Transcriptomic expression

## Abstract

**Background:**

GDSL esterase/lipases (GELPs) play important roles in plant growth, development, and response to biotic and abiotic stresses. Presently, an extensive and in-depth analysis of *GELP* family genes in cotton is still not clear enough, which greatly limits the further understanding of cotton *GELP* function and regulatory mechanism.

**Results:**

A total of 389 *GELP* family genes were identified in three cotton species of *Gossypium hirsutum* (193), *G. arboreum* (97), and *G. raimondii* (99). These *GELPs* could be classified into three groups and eight subgroups, with the *GELPs* in same group to have similar gene structures and conserved motifs. Evolutionary event analysis showed that the *GELP* family genes tend to be diversified at the spatial dimension and certain conservative at the time dimension, with a trend of potential continuous expansion in the future. The orthologous or paralogous *GELPs* among different genomes/subgenomes indicated the inheritance from genome-wide duplication during polyploidization, and the paralogous *GELPs* were derived from chromosomal segment duplication or tandem replication. *GELP* genes in the A/D subgenome underwent at least three large-scale replication events in the evolutionary process during the period of 0.6—3.2 MYA, with two large-scale evolutionary events between 0.6—1.8 MYA that were associated with tetraploidization, and the large-scale duplication between 2.6—9.1 MYA that occurred during diploidization. The cotton *GELPs* indicated diverse expression patterns in tissue development, ovule and fiber growth, and in response to biotic and abiotic stresses, combining the existing *cis*-elements in the promoter regions, suggesting the *GELPs* involvements of functions to be diversification and of the mechanisms to be a hormone-mediated manner.

**Conclusions:**

Our results provide a systematic and comprehensive understanding the function and regulatory mechanism of cotton GELP family, and offer an effective reference for in-depth genetic improvement utilization of cotton *GELPs*.

**Supplementary Information:**

The online version contains supplementary material available at 10.1186/s12864-023-09717-3.

## Background

Cotton is important cash crop to provide raw materials for textile industry. Presently, the tetraploid *Gossypium* spp, upland cotton *G. hirsutum* with the genome constitution (AD1) and sea-island cotton *G. barbadense* with the genome constitution (AD2) are the major cultivars worldwide. The A genome of diploid Asian cotton *G. arboreum* (A2) or grass cotton *G. herbaceum* (A1) and the D genome of *G. raimondii* (D5) are commonly considered as the donor of A subgenome [35, 110, 111] and D subgenome [45, 64, 107, 125] of tetraploid upland cotton and sea-island cotton, despite of the recent study that all A genomes may originate from the common ancestor A0 [47]. As the completion of the genome sequencing and assembly of diploid cotton and tetraploid cotton, more evidences are provided to study the evolutionary relationship among these *Gossypium* spp. [2, 46, 112]. Gene duplication provides the raw material for species evolution, which supplies genomic novelty and complexity through mutational robustness to enhance environmental adaptability [32, 58, 84, 86, 93]. Gene families arise from the continuous expansion of a common ancestor after experiencing a series of evolutionary events, providing important clues to study species evolution that exists within a certain species or between different species. Duplicated genes are usually fractionated, while only a few retained genes will be "neofunctionalization", "subfunctionalization", "back-up compensation", "dosage amplification and stoichiometric balance", and "non-functionalized or pseudogenization" based on the needs of environmental adaptations and bioregulatory networks [20, 30, 33, 59, 67, 79, 104].

*GDSL-type Esterase/Lipase *(GELP) is a superfamily and widely exists in plants, animals, and microorganisms, to hydrolyze various substrates including thioesters, aryl esters, phospholipids, and amino acids by the N-terminal conserved GDSL motif and the Ser-His-Asp/Glu triad catalytic active site [23]. Many plant *GELP* gene families have been identified [91], including *Arabidopsis thaliana*, *Sedum alfredii*, *Oryza sativa*, soybean, cabbage, poplar, grape, sorghum, pecan, and *Brassica napus* with the gene members of 104, 80, 114, 194, 112, 126, 96, 130, 87, and 240, respectively [16, 26, 27, 54, 61, 66, 95, 105]. Evidences have been validated that *GELP*s act as key regulator in plant growth and development. A GDSL lipase *Rice Male Sterility 2* (*RMS2*) plays crucial function in anther and pollen development [126, 127]. Rice *GELPs OsGELP34* and *OsGELP110/OsGELP115* are essential for pollen development by catalyzing different compounds to regulate pollen outer wall development [122, 123]. *Arabidopsis GELP77* is crucial for pollen dissociation and fertility [100, 128]. The *Wilted Dwarf and Lethal 1* (*WDL1*) is required for the formation of cuticle and waxy crystal tissues [80]. *Arabidopsis* GDSL lipase *AtEXL4* and *AtEXL6* are extracellular lipases locating in pollen coat, to perform essential function for effective pollination by generating the lipids that act as important components to mediate the interface of the pollen and the stigma [51, 101, 114]. Mutations of the GDSL lipase *Occluded Stomatal Pore 1* (*OSP1*) caused a significant reduction in leaf wax synthesis and stomatal blockage, increase of epidermal permeability, and decrease of transpiration rate, therefor enhancing the drought tolerance [49, 97]. Maize *ZmMs30* encodes a novel GDSL lipase whose loss of function causes anther cuticle defects, irregular pollen outer wall structure, and complete male sterility [1]. Cabbage lipase (*BnLIP1)* and *sinapoylcholine esterase* (*BnSCE3/BnLIP2)* were involved in the seed germination process [18, 19, 68, 69]. *Arabidopsis GELP7* is a plasma membrane-localized protein and enhanced the saccharification efficiency [85]. Our previous work found that a cotton *GhGELP* participated in ovule and fiber development [72].

*GELP*s are involved in plant stress response and hormone-mediated signaling pathways. *Arabidopsis* *GDSL LIPASE 1* (*AtGLIP1)* regulated the systemic immunity through affect of ethylene-signaling components [60, 77, 55, 56]. *Arabidopsis GDSL1* is a functional extracellular *GELP* and significantly enhance the resistance against *Sclerotinia sclerotiorum* [24]. Loquat *GELP* performs important role in flowering under control of gibberellic acid (GA) [53]. *Dasypyrum villosum DvGELP53* is significantly induced by biotic and abiotic stresses and affects the resistance against barley stripe mosaic virus (BSMV) by influencing the long-distance movement of BSMV [124]. *AtGLIP2* played a key role in plant immune response by negatively regulating auxin signaling [62]. Overexpression of *Li-tolerant lipase 1* (*AtLTL1)* in *Arabidopsis* enhanced the salt tolerance and pathogen resistance under control of salicylic acid (SA) [75]. Cosuppression of Arabidopsis *AtGELP22* and *AtGELP23* promoted the drought tolerance via regulation of the abscisic acid (ABA)-mediated signaling [17]. Rice *OsGLIP1* and *OsGLIP2* are important regulators for disease resistance, with the promoted or inhibited expressions to increase or decrease the resistance for plants in response to bacterial and fungal pathogens [34]. Rice GDSL lipase MHZ11 controlled the root development by decreasing sterol content and participating the ethylene signaling pathway [127]. Apple GELP1 enhanced the resistance to *Colletotrichum gloeosporioides* through an indirect involvement of SA biosynthesis [52]. Overexpression of *Brassica napus BnGDSL1* and *Arabidopsis AtGDSL1* significantly promoted the seed germination rate and improved the seedling growth rate [23–25]. Pepper *CaGLIP1* performed important function in disease resistance and abiotic stress tolerance [43].

Transcriptome sequencing provides important reference for identification of key genes and their genetic function study. Many transcriptomes have been publicly released in cotton, including the expression data of tissue specificity, ovule and fiber development, seed germination, biotic and abiotic stresses, etc., which supply important data to facilitate the study of the cotton *GELP* family genes and their potential functions. Our previous study indicated that a cotton *GELP* is involved in the seed growth [72]. Some individual cotton *GELPs* have been reported to play the roles in the response to drought stress and fiber development [70, 115]. Although the *GELP* family and some GELP protein structures in cotton were reported, and the *GELPs* of *G. arboreum*, *G. raimondii*, *G. hirsutum* were identified [70, 109], while, it is still greatly limited for a holistic, systemic and comprehensive understanding for *GELP* family in cotton. In this study, based on the data of genome sequencing and transcriptomes of three cotton species *G. hirsutum*, *G. arboreum*, and *G. rammondii*, we identified the *GELP* gene families and performed a systemic and comprehensive bioinformatic analysis. The evolution relationship and transcriptional expression of these cotton *GELP* genes were also analyzed, as well as their potential biological functions being predicted. These results provide a comprehensive fundamental information of cotton *GELP* gene family and an important support for understanding the evolution of *GELP* genes, and supply effective references for elucidating the potential molecular regulatory mechanism and for offering candidates for genetic improvement.

## Results

### Identification of *GELP* family genes in *G. arboreum*, *G. raimondii*, and *G. hirsutum*

**Fig. 1:**
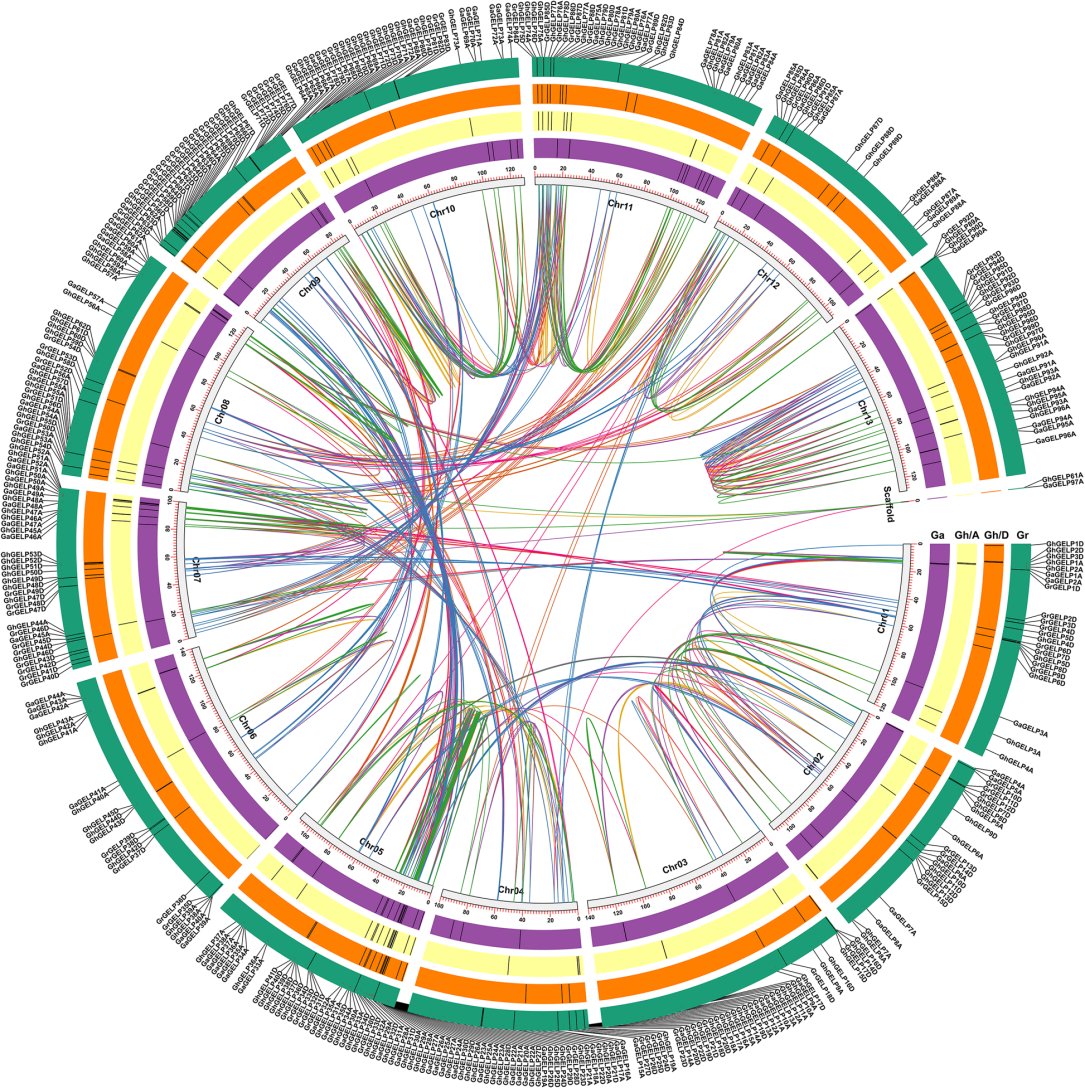
Chromosomal localization of *GELP* genes in three cotton species of *G. hirsutum*, *G. arboreum*, and *G. raimondii*. The green lines show the *GELP* homologous genes between *G. arboreum* and *G. hirsutum* A subgenome; The orange lines represent the *GELP* homologous genes between *G. hirsutum* A subgenome and D subgenome; The purple lines indicate the *GELP* homologous genes between *G. hirsutum* D subgenome and *G. arboreum*; The blue lines denote the *GELP* homologous genes between *G. hirsutum* D subgenome and *G. raimondi*i; The red lines indicate the *GELP* homologous genes between *G. hirsutum* A subgenome and *G. raimondii*; and thebrown lines display the *GELP* homologous genes between *G. arboreum* and *G. raimondii*

The protein sequences of 104 *Arabidopsis* [61] and 114 rice [16] GELPs were used as reference to perform BLASTP by searching the database of COTTONGEN (https://www.cottongen.org/) (Supplementary Table 1), and a total of 389 *GELP*s were finally identified by conservative domain prediction analysis and multiple sequence alignment analysis, including 97 *GaGELPs* in *G. arboreum*, 99 *GrGELPs* in *G. raimondii*, and 193 *GhGELPs* in *G. hirsutum* (Supplementary Table 2), with all the *GELP* sequences listed in Supplementary Table 3. Cotton *GELPs* contained the coding region from 663—1224 bp and encoded the proteins of 221—408 amino acids and of the molecular weight of 24.8 kDa—45.8 kDa (Supplementary Table 4). Of the 389 *GELPs*, 355 included the signal peptide, in which 85 contained both signal peptide and transmembrane regions with the deduced locations in endoplasmic reticulum membrane system and the plasma membrane, and 270 *GELPs* were predicted as secreted enzymes to distribute in extracellular space (Supplementary Table 4). There are 34 *GELPs* to localize in different organelles such as chloroplasts, nucleus, and extracellular space, etc. These *GELP* genes distributed on different chromosomal positions of A or D genomes or subgenomes (Fig. 1).

### Phylogeny, gene structure, and conserved motif analysis of GELP family

**Fig. 2:**
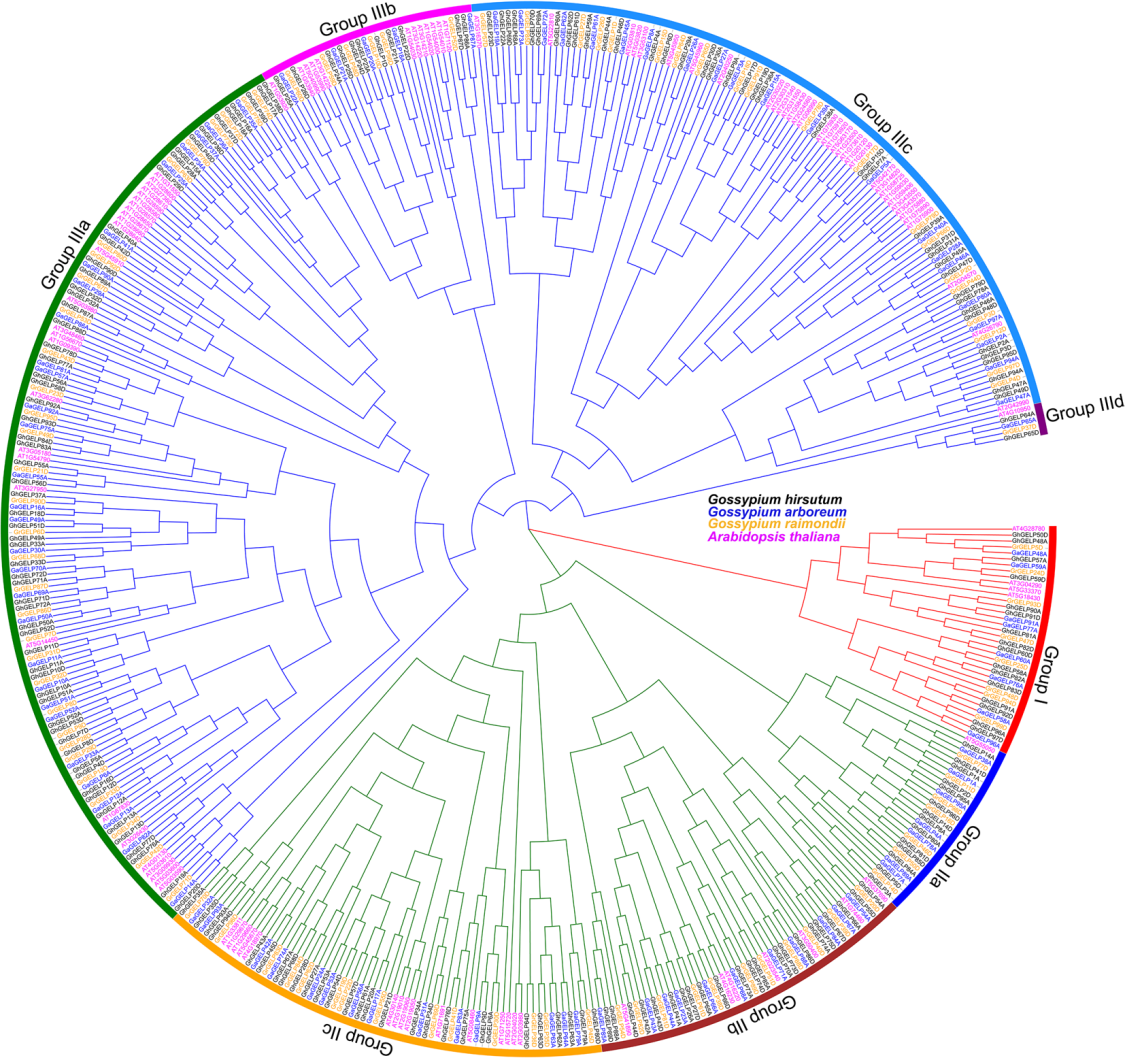
The phylogenetic relationship among GELP families of *Arabidopsis*, *G. hirsutum*, *G. arboreum*, and *G. raimondii*. The GELPs from *G. hirsutum*, *G. arboreum*, *G. raimondii* and *Arabidopsis* were used for phylogenetic tree construction. Different colored lines denote the differnt clusterd groups of Group I—Group III. Diverse colored arc lines reprenent the clustered distrinct groups

The 389 *GELPs* were subjected to the online protein database Pfam and InterPro for conserved motif analysis, which indicated that they contained a conservative characteristic GDSL esterase/lipase domain (Supplementary Table 5). Gene Ontology (GO) analysis showed that they have the molecular function of hydrolase activity acting on ester bonds and the involvement of the biological process of lipid metabolism (Supplementary Table 5). The phylogenetic tree was constructed for the *GELPs* of *G. arboreum*, *G. raimondii*, *G. hirsutum*, and *Arabidopsis*, which indicated that these *GELPs* could be clustered into three groups and eight subgroups, namely Group I, IIa, IIb, IIc, IIIa, IIIb, IIIc, and IIId, respectively (Fig. 2). There existed 2—7 exons in cotton *GELP* genes, and in different phylogenetic groups. There are three cases of changes in gene structure due to deletion or insertion of genomic DNA fragments during the whole genome duplication, resulting in changes in introns or exons of *GELP* genes (Supplementary Figure S1). The *GELP* proteins contained five conserved functional blocks, Block I—V, with the central site of enzymatic activity in Block I, III, and V. The gene structure and protein motif of orthologous or paralogous genes of Group I, IIa, and IIId are relatively conserved during the gene duplication. Some orthologous or paralogous genes lost some motifs or blocks in some subgroups (Supplementary Figure S1). The gene structures and conserved motifs of *GELPs* indicate that large-scale insertion or deletion of genomic DNA fragments occurred in the genome, resulting in chromosomal rearrangement of genomic DNA during cotton evolution, and leading to the neofunctionalization, subfunctionalization, and non-functionalized of these enzymes.

### Spatiotemporal expansion and contraction of the *GELP* family genes during polyploidization of *Gossypium* spp

**Fig. 3:**
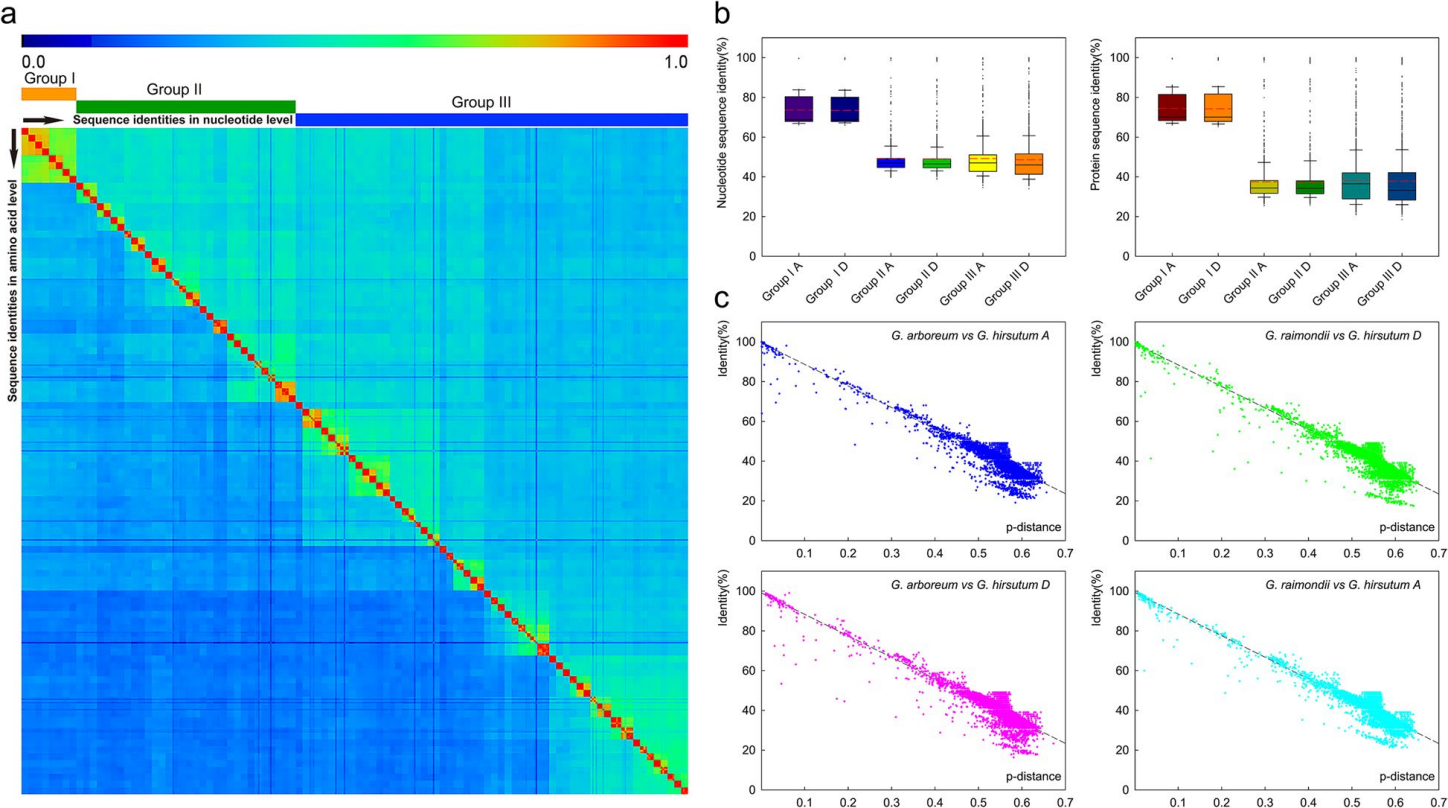
Sequence identity of the *GELP* genes in three cotton species *G. hirsutum*, *G. arboreum*, and *G. raimondii*. **a** Heat-map of sequence identity matrix between the nucleotide and amino acid levels. The color scale at the top of the heat map indicates the level of the sequence identities with light blue and red to represent low and high levels. The data at the diagonal lines are equal to 100%. **b** The identity of the *GELP* genes on the A and D subgenome among the three groups is compared at the nucleotide or amino acid level. **c** The correlation between the sequence identity of *GELP* gene and genetic distance

The matrix of sequence identity at the nucleotide level versus the amino acid level for all cotton *GELP* orthologous or paralogous gene pairs showed that, the sequence identity of nucleotide is significantly higher than that of amino acid. The *GELP* genes could be divided into three major groups according to the differences in sequence identity (Fig. 3a), showing high consistence with the phylogenetic tree (Fig. 2). The orthologous or paralogous gene pairs clustered in same groups from the A or D subgenome indicated similar pattern in nucleotide or protein sequence consistency (Fig. 3a). Group Ia and Id have the least number of homologous gene pairs and the highest sequence identity at both the nucleotide and amino acid levels, while Group IIIa and IIId contained more homologous gene pairs than Group IIa and IId with less sequence identity at nucleotide and amino acid level (Fig. 3b). The relationship between sequence identity and genetic distance (P-distance) for orthologous or paralogous gene pairs displayed that the sequence identity of *GELP* gene pairs from the ancestral diploid genome and the descendant tetraploid genome decreased along with the increase of genetic distance P-distance, showing a large-scale expansion trend, suggesting that the cotton *GELP* family tended to be diversified at the spatial dimension during its evolution (Fig. 3c).

**Fig. 4:**
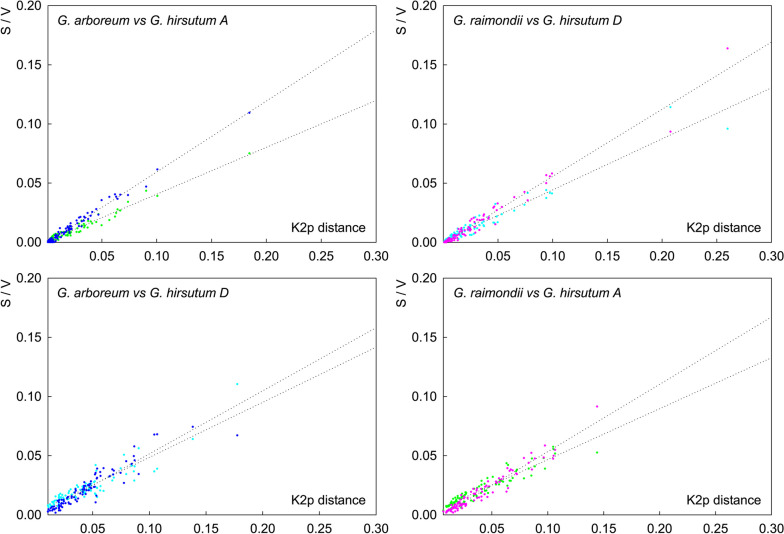
Saturation of base substitutions between nucleotide sequences of orthologous or paralogous gene pairs. Kimura 2-parameter corrected genetic distance is estimated by MEGA-X. S: Transitions; V: Transversions

Gene base substitutions, including base transitions and base transversions that result in silence, missense, nonsense, and termination codon mutation, are major driving forces during gene evolution, leading to structural variation and functional alteration or loss of genes [36, 38, 98, 99]. In order to explore the evolutionary trend of the cotton *GELP* family genes at the time dimension, the base substitution saturation between orthologous or paralogous gene pairs that evolved from ancestral diploid to descendant tetraploid was determined by calculation (Fig. 4). The results showed that the base transition rate was higher than the base transversion rate among orthologous gene pairs, and the base transversion rate was higher than the base transition rate among paralogous gene pairs. Along with the increase of the corrected genetic distance (K2p-distance), the base transition rate or base transversion rate between orthologous or paralogous gene pairs showed a linear upward tendency, which was far from reaching the saturation state (Fig. 4). These data indicated that the evolution degree of *GELPs* in cotton is conservative to a certain extent, and there is still a potential evolutionary expansion trend.

**Fig. 5:**
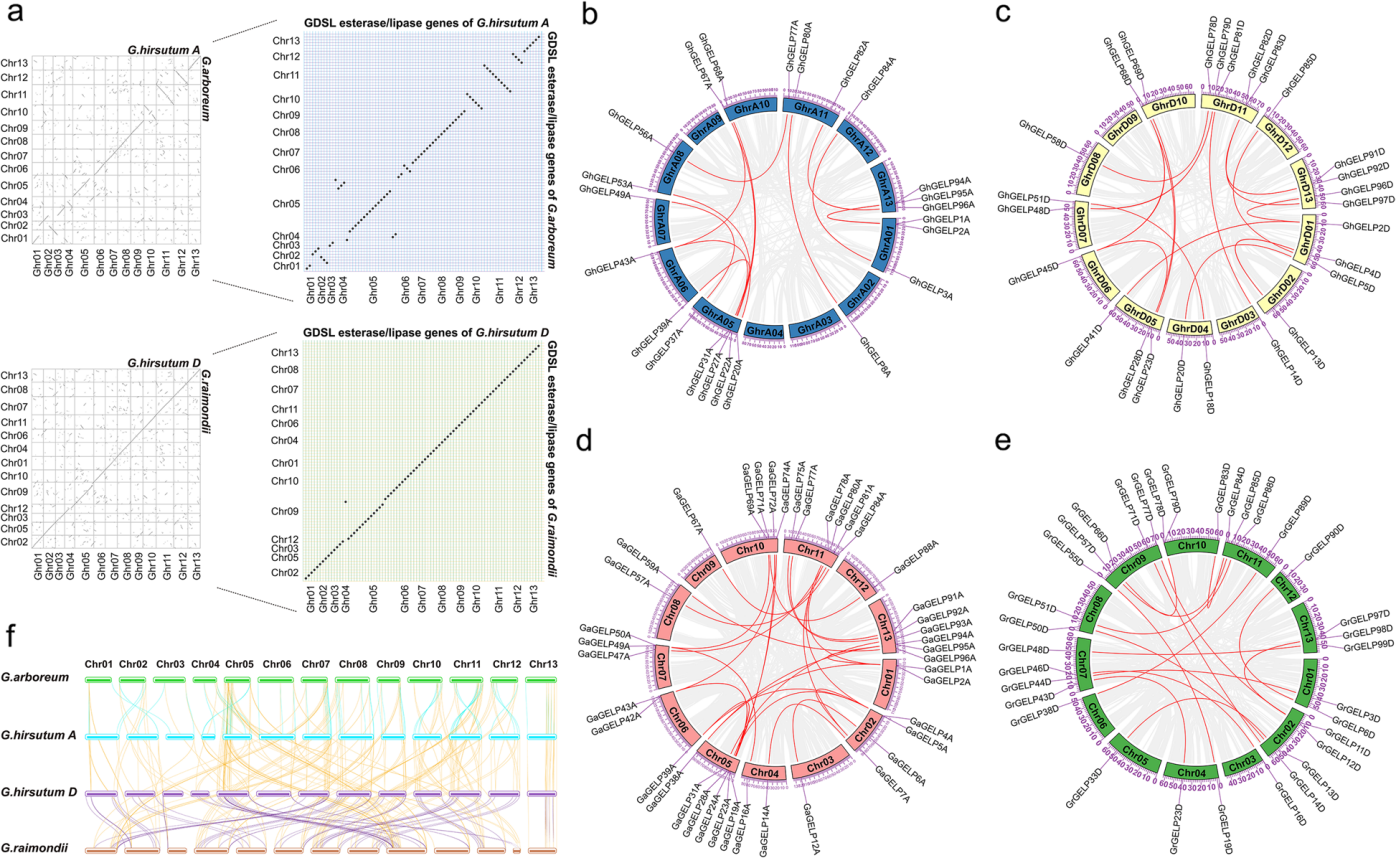
WGD/segmental duplication event of *GELP* genes in *G. arboreum*, *G. raimondii*, and *G. hirsutum*. **a** Dotplot of *GELP* homologous gene pairs in subgenome. **b** The collinear relationship of *GELP* genes in the A subgenome of *G. hirsutum*. **c** The collinear relationship of *GELP* genes in the D subgenome of *G. hirsutum*. **d** The collinear relationship of *GELP* genes in *G. arboreum*. **e** The collinear relationship of *GELP* genes in *G. raimondii*. **f** The collinear relationship of *GELP* genes among different subgenomes of *Gossypium* spp. The *GELP* collinear gene pairs were presented by red line in b—e. The *GELP* collinear gene pairs of different subgenomes were indicated by different colored lines in f

The multi-copy gene families record all evolutionary events that have occurred since ancient times and are produced by the continuous expansion of a common ancestor after a series of evolutionary events [22], and their expansions are mainly through whole genome duplications (WGD), small-scale fragment duplications, tandem duplications, proximal duplications, transposon-mediated duplications, lateral gene transfer, and retroduplications caused by the "copy and paste" mechanism during reverse transcription [79, 82, 84, 59]. To understand the duplication events of the orthologous or paralogous genes of the *GELP* family genes during the evolution from diploid to tetraploid, the colinearity within subgenomes was analyzed, and the results indicated that, the collinearity between the diploid A/D genome and the tetraploid A/D subgenome showed different degrees of large-scale chromosome segment duplication events between the subgenomes (Fig. 5a). The collinear relationship between *GELP* homologous genes is consistent with the collinear relationship between the diploid genome and the tetraploid subgenome (Fig. 5a), indicating the expansion of cotton *GELP* family genes is mainly related to the large-scale duplication of chromosome segments that occurred during polyploidization. The collinear relationship of *GELP* paralogous genes within each subgenome showed that more collinear paralogous gene pairs within diploid A/D genome than within tetraploid A/D subgenome were observed (Fig. 5b—f), suggesting a large-scale insertion or deletion of chromosome fragments in cotton genome occurred during tetraploidization, resulting in the dislocation of orthologous genes and the generation of non-collinear paralogous genes. The orthologous or paralogous *GELP* genes among different subgenomes were inherited from genome-wide duplication during polyploidization, whereas paralogous genes within subgenomes were derived from chromosomal segment duplication or tandem replication. The evolution of cotton genome is affected by natural selection pressure, mutation, and polyploidization, leading to chromosome rearrangement and large-scale insertion or deletion of chromosome fragments, which generates some non-collinear orthologous or paralogous genes within or between subgenomes (Supplementary Table S6).

**Fig. 6:**
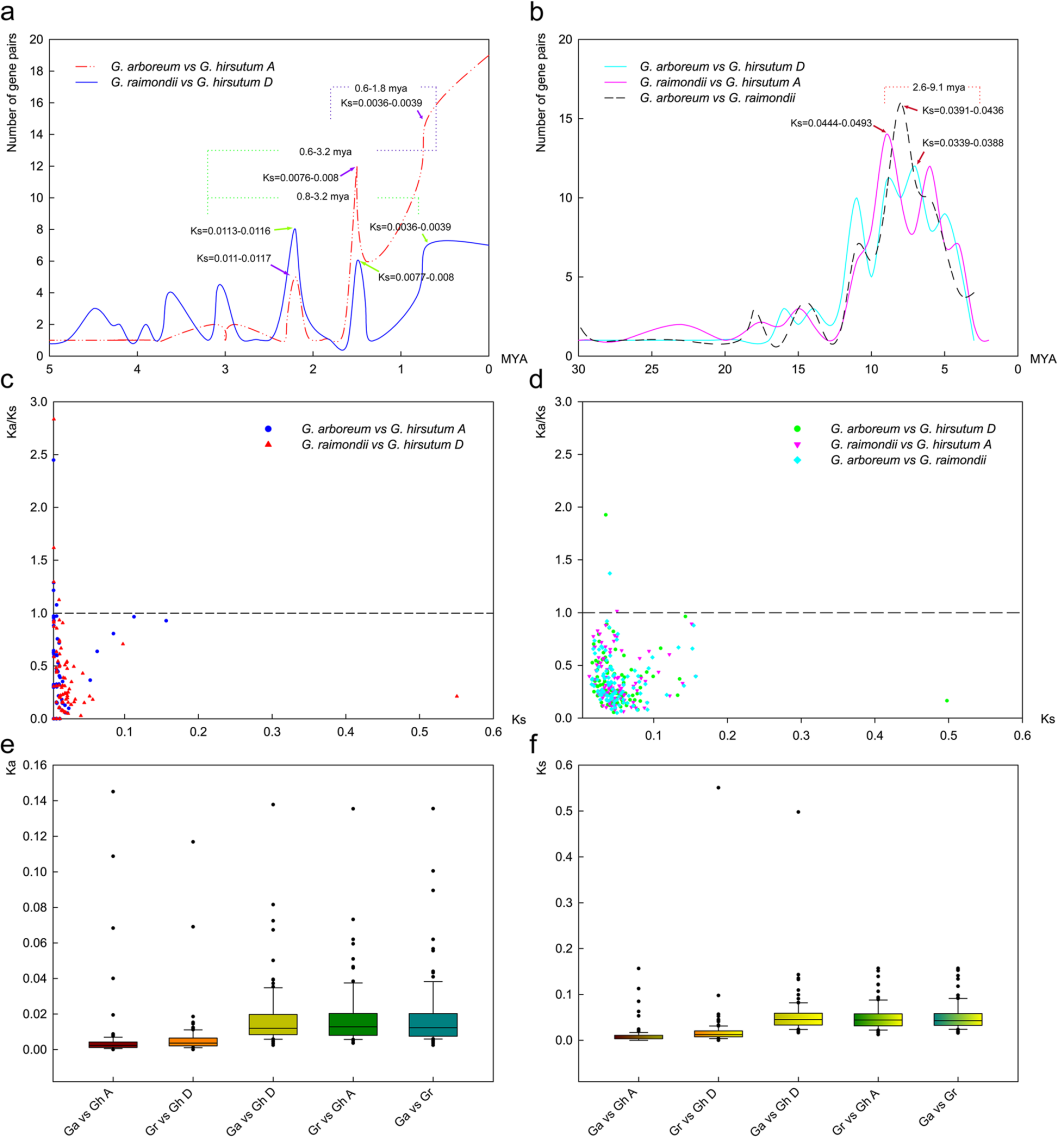
Evolutionary event analysis of *GELP* genes in *G. arboreum*, *G. raimondii*, and *G. hirsutum*. Evaluation of evolutionary events among orthologous *GELPs* **a** and paralogous *GELPs* **b**; Evolutionary selection pressure among orthologous *GELPs* **c** and paralogous *GELPs* **d**; The rate of non synonymous substitution (Ka) **e** and synonymous substitution (Ks) **f** among *GELPs* in *G. arboreum*, *G. raimondii*, and *G. hirsutum*

To assess the evolutionary events retained in cotton *GELP* family genes, the rate of heritable variation during evolution was converted to a yardstick of evolutionary time to discuss possible evolutionary events. The results showed that *GELP* genes in the A/D subgenome of cotton underwent at least three large-scale replication events in the evolutionary process during the period of 0.6—3.2 MYA, of which the latest two large-scale replication events occurred during the period of 0.6—1.8 MYA, and the oldest large-scale replication event occurred during the period of 2.6—9.1 MYA. Thus, the tetraploidization process of the cotton led to two large-scale evolutionary events of *GELP* family genes during 0.6—1.8 MYA, and the oldest large-scale duplication occurred during 2.6—9.1 MYA was a symbol of diploidization, and a large-scale duplication that occurred on the A/D genome during 0.8—3.2 MYA followed diploidization and before tetraploidization (Fig. 6a—b).

The rate of heritable variation depends on Ks and the Ka of the bases in the gene sequence within a certain period of time, and the ratio of Ka/Ks is used to reflect the evolutionary selection pressure on biological macromolecules during evolution, with Ka/Ks > 1 or < 1 to indicate positive selection or purification selection. To discuss the selection pressure of *GELP* family genes in cotton during the evolution, the base substitution rate (Ka and Ks) within the sequences from orthologous or paralogous gene pairs were analyzed (Fig. 6c—f, Supplementary Table 7). There are eight pairs of orthologous gene pairs and three pairs of paralogous gene pairs in the cotton *GELP* family that are suffering from positive selection pressure (Ka/Ks > 1). The Ka/Ks rates between orthologous gene pairs are far lower than those between paralogous gene pairs (Fig. 6c—d, Supplementary Table 7).

### Functionally enriched *GELPs* regulate growth and stress response in cotton

To investigate the potential functions of cotton *GELP* family genes involving in fiber growth, tissue development, and biotic and abiotic stress response, the FPKM values of *GELP* genes in five sets of upland cotton transcriptome data that have been publicly released were obtained for expression level analysis of the *GELP* genes. The results showed that, the expression profiles of *GELP* genes in cotton tissues of roots, stems, leaves, receptacles, petals, stamens, pistils, and calyx could be clustered into seven clusters (Cluster 1, 2a, 2b, 2c, 2d, 3a, and 3b). The expression levels of *GELP* genes in Cluster 1, 3a, and 3b were significantly higher than in other clusters, and six *GELPs* in Cluster 1 were more accumulated in each tissue than those in Cluster 3a and 3b. In addition, the *GELPs* in Cluster 2a, 2b, and 2c have higher expressions in stamens, leaves, and stems, respectively (Supplementary Figure S2, Supplementary Table 8). These results indicated that the *GELPs* of upland cotton not only gradually alienated specific functions but also differentiated the corresponding tissue specificity during the evolution.

During the whole growth period of fiber development (−3 to 25 dpa), the expression patterns of *GELP* genes were clustered into seven clusters including Cluster 1, 2a, 2b, 3, 4a, 4b, and 4c. The *GELPs* of Cluster 1 showed the most accumulated expressions in fiber initiation and elongation stages (−3 to 15 dpa), Cluster 3 *GELPs* indicated significant increased expression in fiber secondary thickening stage (20 to 25 dpa), Cluster 2a and 2b *GELPs* have a relative higher expressions in fiber elongation and secondary thickening stages (3 dpa to 25 dpa) and in fiber initiation and elongation stages (−3 to 10 dpa), while the *GELPs* of Cluster 4a, 4b, and 4c were expressed at low levels throughout the whole period of fiber growth (Supplementary Figure S3, Supplementary Table 9), suggesting these *GELPs* clustered in different groups may perform possible different functions involving in fiber growth and development.

**Fig. 7:**
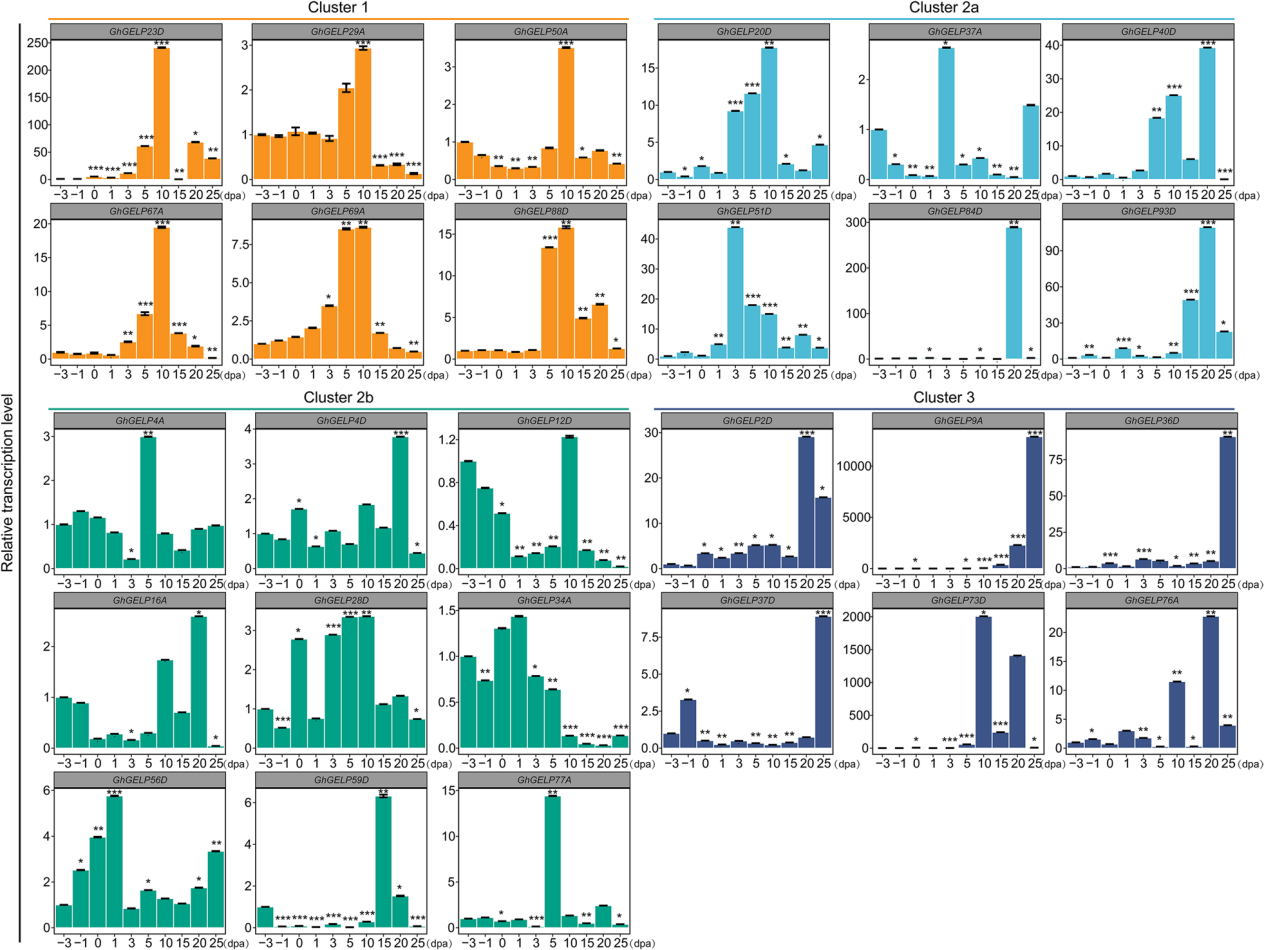
Real-time quantitative PCR (RT-qPCR) detection analysis of *GhGELPs* expressions during fiber development stages. The ovules and fibers of different developmental periods of −3, −1, 0, 3, 5, 10, 15, 20, and 25 dpa were collected for RNA extraction and cDNA synthesis that were then used as templates for RT-qPCR with *GhUBQ7* (DQ116441.1) as inter control for normalization. Error bars were calculated by three independent experiments. Significant difference was calculated by Student’s *t*-test using the data of −3 dpa as control, with *, **, and *** to represent *p* < 0.05, 0.01, and 0.001 respectively

To verify the expression reliability of these *GhGELP* genes during fiber development, 27 *GhGELPs* locating in Clusters 1, 2a, 2b, and 3 that indicated significant accumulations were selected for real-time quantitative PCR (RT-qPCR) experiments. The results showed that the relative expression levels of these *GhGELPs* by qPCR indicated similar tendency and high consistence with that by transcriptomes during the whole fiber development periods (Fig. 7). The expressions of 6 *GhGELPs* in Cluster 1 displayed the most significant enrichments in the period of −3 to 15 dpa, especially in 5- and 10-dpa fast elongating fibers, with the *GhGELP23D* and *GhGELP67A* over 600-fold increase in 10-dpa fibers, providing their possible significant function for fiber elongation. The *GhGELPs* of Clusters 2a and 2b demonstrated a relative higher levels during the whole period of fiber development, with the major accumulations for Cluster 2a *GhGELPs* in elongation and secondary wall thickening stages and most Cluster 2b *GhGELPs* in initiation and elongation stages. Cluster 3 *GhGELPs* were predominantly expressed during secondary wall thickening stage, with approximate 150-fold increase for *GhGELP9A* in 25-dpa fibers and over 20-fold increase for *GhGELP73D* in 10-dpa fibers, showing their potential role in fiber wall thickening (Fig. 7, Supplementary Figure S3, Supplementary Table 9).

During the different stages of seed germination, cotyledon growth, and root development, *GELPs* indicated diverse expression profiles with six clustered groups of Cluster 1, 2a, 2b, 3, 4a, and 4b. Cluster 1 *GELPs* expressed the most significant accumulation in all stages, Cluster 2a *GELPs* mainly expressed in cotyledon growth, Cluster 2b *GELPs* showed a relative higher levels in all stages, and Cluster 3 *GELPs* indicated a moderate expressions in root development (Supplementary Figure S4, Supplementary Table 10), implying these *GELPs* may perform different roles in tissue development. The *GELPs* displayed diverse expression features with typical four clustered groups of Cluster 1—4 in response to abiotic stress including cold, hot, salt, and drought. Cluster 1 *GELPs* were significantly expressed under these four stress treatments for different time points, and Cluster 2 *GELPs* showed a relative higher expressions (Supplementary Figure S5, Supplementary Table 11), suggesting these accumulated *GELPs*, especially the 14 genes of Cluster 1, are important response regulators for plants in response to abiotic stress. Verticillium wilt caused by *Verticillium dahliae* is a serious disease during cotton whole growth stage to greatly reduce the quality and yield of fibers [71]. Of the four classified clusters (Cluster 1—4), Cluster 1 and 2 *GELPs* showed significant accumulation and moderate expression respectively against *V. dahliae* treatment for different time points (Supplementary Figure S6, Supplementary Table 12), which indicated that these *GELPs* are key factors for the response process to *V. dahliae* and can be further utilized as candidates for genetic improvement.

**Fig. 8:**
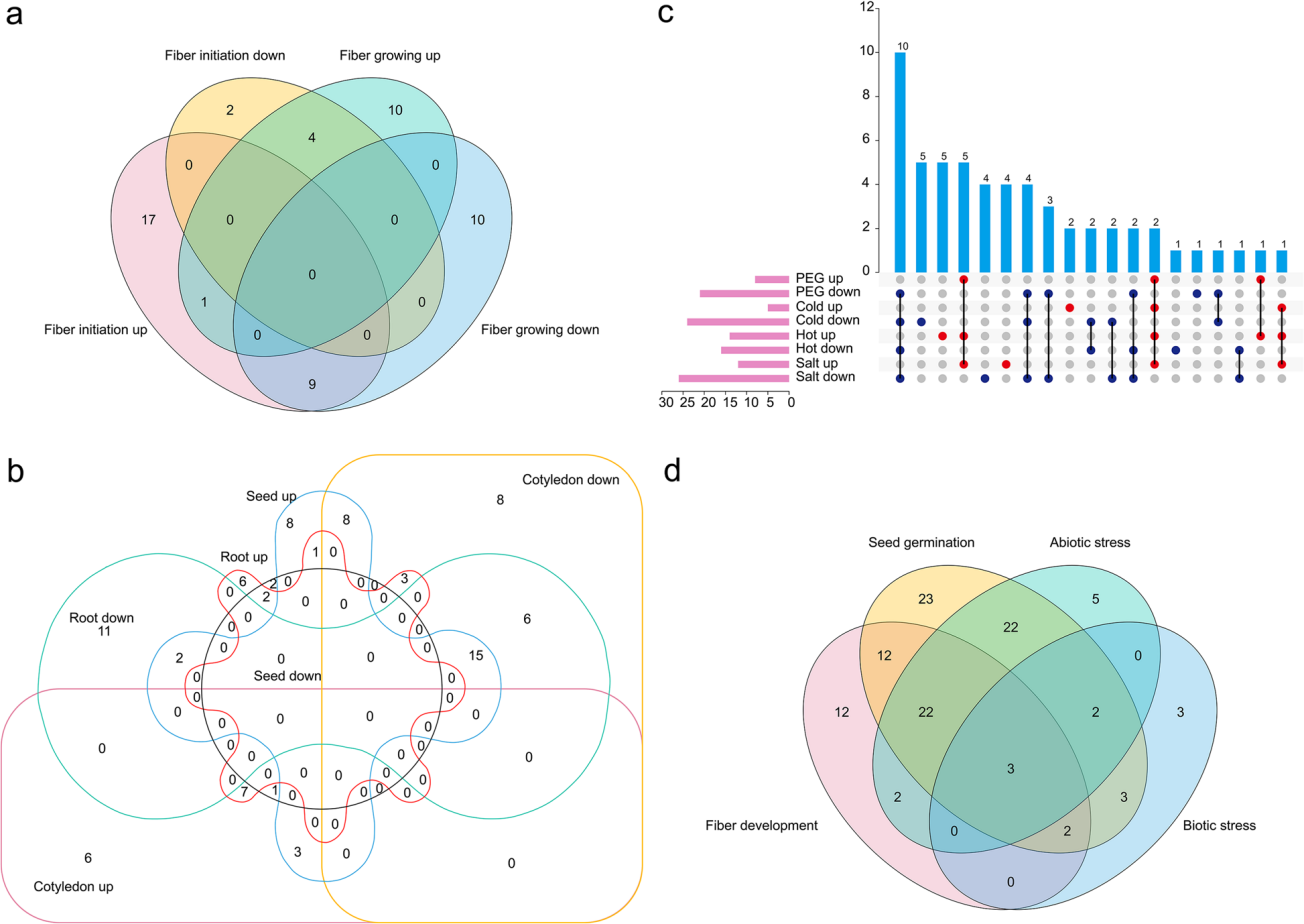
Co-expression analysis of differentially expressed *GELP* genes. Co-expression analysis of differentially expressed *GELP* genes during the processes of fiber development **a**, seed germination **b**, diverse abiotic stresses **c**, and different growth, development, and stress **d**

The differential expression and co-expression analysis of *GELP* genes can more clearly elucidate the relationship between expressions and functions. During fiber development stages of fiber initiation (−3, −1, 0, 1, and 3 dpa) and fiber growth (5, 10, 20, and 25 dpa), a total of 27 and 6 *GELPs* were significantly up-regulated and down-regulated during fiber initiation stage, and 15 and 19 *GELPs* were significantly up-regulated and down-regulated during fiber growth stage, respectively. Of the up-regulated *GELPs*, *GhGELP24D* was significantly accumulated in both stages, while 17 and 10 *GELPs* indicated specific increased expressions in the stages of fiber initiation and fiber growth, respectively (Fig. 8a; Supplementary Figure S7; Supplementary Tables 13, 14), showing these *GELPs* crucial functions in fiber development.

During seed germination and tissue development process, a total of 37 and 5 in seed germination, 17 and 40 in cotyledon development, and 19 and 34 in root development showed significantly increased and decreased expression levels, respectively. Of the up-regulated *GELPs*, there were 8 in embryos and 6 in cotyledons or roots expressed tissue-specificity, and 7 *GELPs* were co-expressed in cotyledons and roots, interestingly, 4 *GELPs* of *GhGELP1A*, *GhGELP2D*, *GhGELP21A*, and *GhGELP26D* are co-expressed in seeds and cotyledons or roots, indicating their close important connection with tissue development. Of the down-regulated *GELPs*, 2, 8, and 11 *GELPs* were specifically expressed in seeds, cotyledons, and roots, respectively. Of which, *GhGELP10D*, *GhGELP24A*, and *GhGELP25D* were co-expressed in embryos as down-regulated genes, with different up-regulated expressions of *GhGELP25D* in cotyledons and of *GhGELP10D* and *GhGELP24A* in roots (Fig. 8b; Supplementary Figure S7; Supplementary Tables 15, 16, 17), showing a potential different regulation mechanism for tissue development.

During the response process to the four abiotic stresses of drought, salt, hot, and cold, two *GELPs* (*GhGELP7A* and *GhGELP33D*) in four treatments, six (*GhGELP5A*, *GhGELP8D*, *GhGELP11D*, *GhGELP15D*, *GhGELP58A*, and *GhGELP35D*) in three treatments, and one (*GhGELP76A*) in two treatments were significantly co-expressed with up-regulated levels. Of the down-regulated *GELPs*, ten in four treatments, six in three treatments, and nine in two treatments, were discovered as co-expressed *GELPs*, respectively (Fig. 8c; Supplementary Figure S7; Supplementary Tables 18, 19, 20, 21). When the co-expression analysis of *GELPs* involved in the four processes of fiber growth, seed germination, abiotic stress response, and biotic stress response (*V. dahliae* treatment) was performed, the results showed that, 39 *GELPs* in two processes, and three in four processes, were discovered (Fig. 8d; Supplementary Figure S7; Supplementary Table 22), which suggest that these different *GELPs* may have consistent important functions in different processes.

**Fig. 9:**
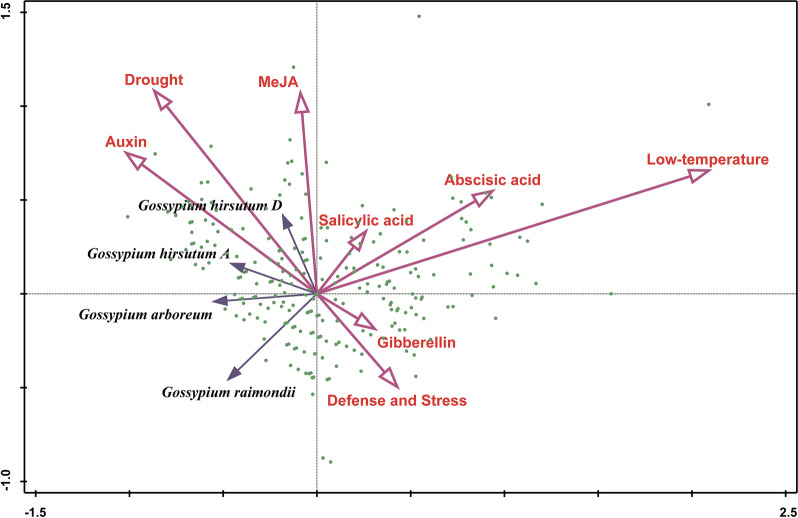
Redundancy analysis of the putative regulatory *cis*-elements of the promoters of *GELP* genes in different cotton genomes/subgenomes. The green dots denote the *GELPs*, the pink arrows indicate the environmental factor varibale, and the blue arrows represent the species factor variable

An important indicator for assessing the function of genes that are involved in growth and development and stress response is the *cis*-acting elements in the promoter region [42, 103]. The 2000-bp sequences upstream of the initiation codon of 389 *GELP* members were subjected for prediction analysis of the *cis*-acting elements responding to plant hormone and environmental stress (Supplementary Figure S8, Supplementary Table 23). All *GELP* gene promoters contain the basic transcription elements such as CAAT-box and TATA-box, and almost all *GELP* gene promoters include the *cis*-acting elements that respond to plant hormone and environmental stress containing of phytohormone response elements to ABA, methyl jasmonate (MeJA), SA, GA, and auxin, and of stress response elements to drought, low-temperature, and defense (Supplementary Figure S8, Supplementary Table 23). There are 48.6% and 56.1% *GELP* gene promoters include the ABA and MeJA response elements, showing their potential role in plant growth and stress response. To explore the conservation, preference, and difference of *cis*-acting elements distributing in the promoter region of the cotton *GELP* genes during the evolution from the ancestral diploid to the descendant tetraploid, the redundancy analysis of *cis*-acting elements of the promoter regions of *GELP* homologous genes in different subgenomes was performed (Fig. 9). There is 22.4% *GELP* gene promoters that have auxin-responsive elements (ARE), which are the most conservative ancient responsive elements due to the significant positive correlation between ARE and all subgenomes (Fig. 9). Drought response elements (DRE) are also the ancient conserved response elements, and are significantly associated with the other three genomes except for *G. raimondii*; while the defense and stress response elements are positively correlated with the *G. raimondii* genome, indicating that cotton *GELP* gene functions tend to be diversification and the mechanisms of defense and stress response evolve as a hormone-mediated manner.

**Fig. 10:**
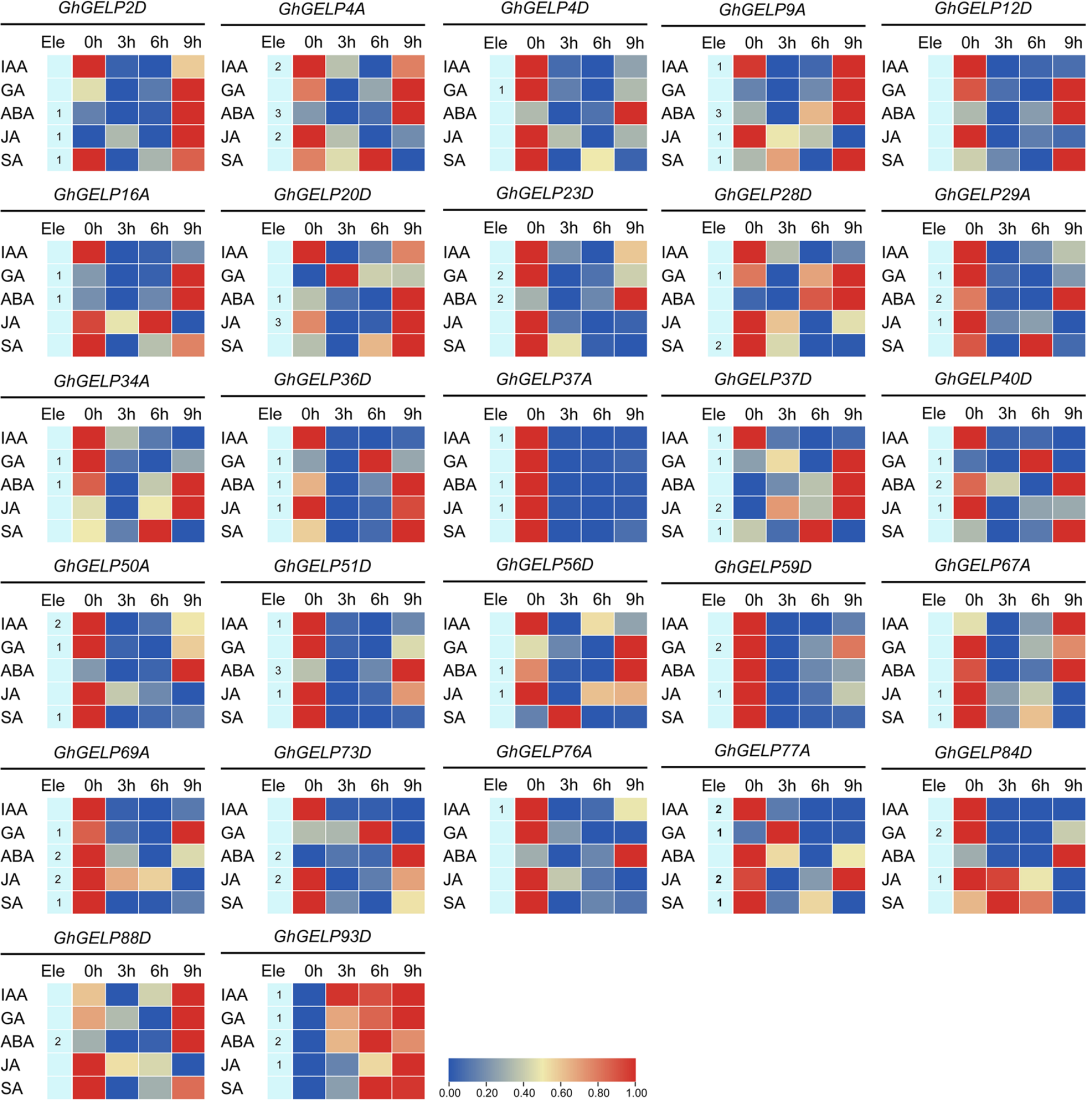
RT-qPCR-based heat-map of *GhGELPs* expression levels under treatments of plant hormone. Cotton leaf materials were treated by the plant hormones IAA, GA, ABA, JA, and SA for 0, 3, 6, and 9 h, which were collected for RNA extraction and cDNA synthesis that were then used as templates. *GhUBQ7* (DQ116441.1) was used as internal reference for normalization. Three independent experiments were performed. “Ele” denotes the statistics number of corresponding *cis*-elements that existed in the promoters of *GhGELPs*. The heat-maps were generated by TBtools

Regarding the important role of plant hormone in cotton growth and development [46], and the existing plenty of hormone responsive *cis*-elements in *GhGELPs* promoters (Supplementary Figure S8, Supplementary Table 23), to investigate the correlation between the *GhGELPs* expressions and plant hormone, RT-qPCR detections of selected *GhGELPs* under plant hormone treatments (IAA, GA, ABA, JA, and SA) were performed. These *GhGELPs* indicated diverse expression profiles under different phytohormone treatments (Fig. 10). *GhGELP9A*, *GhGELP67A*, *GhGELP88D*, and *GhGELP93D* were significantly up-regulated after IAA treatment, of which *GhGELP67A* and *GhGELP88D* were also co-expressed in fiber development, seed germination, and abiotic stress response. Of the 16 GA-induced *GhGELPs*, eight contained putative GA responsive *cis*-acting element, and 10 *GhGELPs* indicated accumulations during fiber development and seed germination and two *GhGELPs* (*GhGELP20D* and *GhGELP28D*) showed up-regulated expression in response to abiotic stress. After ABA treatment, 15 out of the 23 significantly induced *GhGELPs* included putative ABA responsive *cis*-acting element. A total of 8 and 13 *GhGELPs* were significantly induced under JA and SA treatment, respectively. Among them, six *GhGELPs* contained putative JA responsive *cis*-acting elements and two *GhGELPs* had SA responsive *cis*-acting elements. These results showed that there is a close link between *GhGELPs* expression and phytohormone regulation possibly controlled by the existed corresponding *cis*-acting elements in the promoters.

## Discussion

The multifunctional *GELPs* play important roles in participating in the regulation of biological growth and development and stress resistance-related life activities. A total of 389 *GELP* members were identified in three *Gossypium* species *G. arboreum, G. raimondii*, and *G. hirsutum*, most *GELPs* are hydrophilic proteins and contain signal peptide sequences at the N-terminus with the predicted location in the extracellular space (Fig. 1, Supplementary Table 4), suggesting their potential function as secreted proteins. Some *GELPs* are predicted with the locations in nucleus, plasma membrane, organelle and endomembrane system (Supplementary Table 4), indicating that they may be the basic elements in the growth process of plant cells. A total of 104 and 114 *GELPs* were identified in *Arabidopsis* and rice with classification into different groups [16, 61]. The cotton *GELPs* distributing on different chromosomes can be clustered into eight groups (Fig. 2), with the *GELPs* classified into same groups to share similar gene structure and conserved motifs (Supplementary Figure S1) and thus to perform consistent biological functions.

Of the four *Arabidopsis GELPs* in Group I, the transgenic plants overexpressing *AtCUS1 *(*cutin synthase 1*) enhanced the salt tolerance and promote the germination, flowering, and fruiting under salt conditions [75]. *AtCUS2* is a downstream target of the class I TEOSINTE BRANCHED 1, CYCLOIDEA, PCF (TCP) transcription factor TCP15 and AP2/EREBP transcription factor SHINE1 [10], and affects the formation of plant epidermal ridge. Co-silencing of *AtCUS1* and *AtCUS2* resulted in severe alteration of floral organ development [92, 44]. *AtCUS3* is a Guard-cell-enriched GDSL lipase (GGL26) to influence the opening, density, and morphology of stoma possibly under regulation of GA signal [6, 11, 113]. *AtCUS4* is controlled by drought and Brassinosteroids [9, 37, 120]. AtCDEF1 (CUTICLE DESTRUCTING FACTOR 1) is required for pollen development by degrading stigma cuticle to enhance the penetration of pollen tubes into stigma [87, 96]. In this work, the expression profiles of *GELP* genes in eight different cotton tissues were divided into seven clusters with diverse expression features in different tissues (Supplementary Figure S2), showing their important role in tissue development. During the whole growth process of fiber development, different stages of seed germination, cotyledon growth and root development, and diverse periods in response to abiotic and biotic stress, the *GELPs* indicated differential expression patterns with different clusters (Supplementary Figures S3, S4, S5, S6), suggesting these *GELPs* in different groups may perform possible different functions involving in these processes. The *Arabidopsis GELPs* have been reported to regulate the biosynthesis of plant cuticle that acts as protective layer composed of cutin and wax locating on the outer epidermal cell wall of plants, which enables the important function for plants survival, development, and interaction with environment factors [94].

Three *Arabidopsis GELPs* (AT1G74460, AT2G23540 and AT5G37690) of Group IIb were strongly correlated with endodermal suberization under auxin regulation, and involved in the formation of cutin polymers [102]. The expression levels of some cotton *GELP* members of Group IIb were changed significantly in response to *V. dahliae* infection (Supplementary Figure S6). Six *Arabidopsis* members of Group IIc were up-regulated by gibberellin in *gal-3* mutants, GGL5 plays a role in the vascular system and is involved in phloem-mediated long-range signaling to regulate local defense responses to biotic and abiotic stresses by ethylene signaling [4, 7, 39]. Loss-of-function mutant *ggl6* indicated enhanced susceptibility to *S. sclerotiorum*, overexpression of *GGL6* increased seed germination rate and lipid precursor phosphatidic acid content [23–25]. GGL7 participates in stomatal movement and is closely connected with drought tolerance [113, 119]. At1g71250 and AT1G71691 are reported to perform important function in fatty acid metabolism regulated by the bHLH and MYB transcription factors [29, 57]. *AtLIP1* (AT5G45670) could promote seed germination potential under control of the HD-ZIP transcription factor ATML1 to bind to the promoters in the regulation by GA signaling [12, 89]. Rice GER1 (CONTAINING ENZYME RICE 1) is important regulator for coleoptile elongation [8, 88]. The GELP member SFARs are crucial for stress response, seed germination, seedling growth through affecting the metabolism process of fatty acid, choline esters, and xyloglucans [14, 21, 48, 50, 73].

Genetic evidences have been studied that *GELPs* in Group IIIb are mainly reflected in stress resistance and antibacterial activity. Group IIIb has five *GELPs* in *Arabidopsis* including AT5G40990 (*GLIP1*), AT1G53940 (*GLIP2*), AT1G53990 (*GLIP3*), AT3G14225 (*GLIP4*) and AT1G53920 (*GLIP5*). GLIP1 can directly destroy the integrity of fungal spores and enhance local and systemic resistance in plants through ethylene signal transduction pathways [55, 56, 60, 77]. The ethylene-regulated transcription factor WRKY33 can directly bind to *GLIP* promoter to increase the resistance of plants to *Botrytis cinerea* [5, 41, 118]. GLIP2 also showed strong lipase and antibacterial activity to inhibit the germination of fungal spores to contribute to pathogen defense under a negative regulation by auxin [62]. *GLL* genes of Group IIIb can enhance the resistance by stabilizing the structure of endoplasmic reticulum protein polymer of the defense system [74, 116, 117]. These reports indicated the important role of GELPs in response to biotic stress through the powerful lipase activity of defense system via the hormone signal transduction pathways. The cotton *GELPs* of Cluster IIIa and IIIb also showed significant accumulation (Supplementary Figure S6), considering the existing *cis*-elements of hormones (Supplementary Figure S8, Supplementary Table 2^3^), suggesting their important function in response to *V. dahaliae* through the associated regulation mechanism by a hormone manner.

The analysis of sequence consistency and base substitution saturation between homologous gene pairs of cotton *GELPs* indicated that the evolution of *GELPs* at spatial dimension tends to be diversified and conservative at time dimension during the evolution from ancestral diploid to progeny tetraploid (Fig. 3). There is still a potential evolutionary expansion trend over time, and the demonstration of this reasoning is also reflected in the clustering and branching of the phylogenetic tree, the differences in gene structure, the conservatism of amino acid sequences, and the expression levels involved in different biological functions of *GELP* family members. The collinear relationship between different subgenomes of cotton *GELP* genes indicated that the expansion of the family genes is mainly related to replication events within the diploid genome, but not to replication events within the tetraploid subgenome (Fig. 5). After the genome is doubled, evolutionary events such as large-scale insertion or deletion of chromosome fragments within the genome occur in the tetraploid A/D subgenome, resulting in the misalignment of homologous genes to produce non-collinearity paralogous genes. The essence of species evolution is the process of changing the frequency of population genes on the basis of natural selection or the accumulation of neutral mutations, and the change of population gene frequency is mainly caused by the genetic variations from intra-species mutations and gene recombination [3, 76, 78]. The *GELP* genes on the A/D subgenome of cotton between 0.6—3.2MYA has undergone at least three large-scale replication events during evolution, the most recent two large-scale replication events occurred in 0.6—1.8 MYA, and the oldest large-scale replication event occurred between 2.6—9.1 MYA (Fig. 6), indicating that the two large-scale evolutionary events of *GELP* family genes between 0.6—1.8 MYA were associated with tetraploidization, and the large-scale replication between 2.6—9.1 MYA occurred during diploidization, while the large-scale replication between 1.8—3.2 MYA occurred in the A/D genome of after diploidization but before tetraploidization of cotton. This is consistent with the latest research results that the divergence time between the genome of *G. arboreum* and the A subgenome of *G. hirsutum* was about 1 MYA, and the divergence time between the genome of *G. raimondii* and the D subgenome of *G. hirsutum* was about 1.6 MYA, and the divergence time between *G. arboreum* genome and *G. raimondii* genome was 4.8 MYA [13, 15, 47].

*GELP* genes can strongly participate in both the tissue growth and the systemic acquired immunity or local immune defense system of plants, possibly though the presence of some participants accompanied by hormone signal pathways. The cotton *GELPs* contained diverse hormone responsive elements in the promote regions and displayed different expression patterns in tissues and response to stresses (Figs. 7, 8, 10), suggesting their potential regulation mechanism under control of hormone. *GEPLs* are a kind of ubiquitous multifunctional lipase and perform the biological functions that involved in multiple biological processes and diverse signal pathways, the further molecular regulatory mechanism and genetic validation are still limited and needed to be investigated.

## Conclusions

We identified 389 *GELP* family genes in three cotton species of *Gossypium hirsutum*, *G. arboreum*, and *G. raimondii*, and their gene structure, conserved motif, and *cis*-element component were analyzed. Evolutionary event of the *GELP* family genes was also investigated, showing their diversification at the spatial dimension and certain conservation at the time dimension, with a trend of potential continuous expansion in the future. These cotton *GELPs* indicated diverse expression patterns in tissue development, ovule and fiber growth, and in response to biotic and abiotic stresses, displaying their potential multifunctions and regulatory mechanism in a hormone-mediated manner. Our results provide a systematic and comprehensive understanding the function and regulatory mechanism of cotton *GELP* family, and offer an effective reference for in-depth genetic improvement utilization of cotton *GELPs*.

## Methods

### Identification of cotton *GELP* genes

The protein sequences of 104 *A. thaliana AtGELPs* [61] from The Arabidopsis Information Resource (TAIR) database (https://www.arabidopsis.org) and 114 *O. sativa OsGELPs* [16] from Rice Genome Annotation Project (RGAP) database (http://rice.plantbiology.msu.edu) were used as query sequences to obtain cotton *GELP* family members by matching the whole genome data files of the diploids *G. arboreum* (A2) [28, 65] and *G. raimondii* (D5) [81, 106, 108], and the tetraploid *G. hirsutum* (AD1) [45, 64, 107, 125] from the CottonGen database (https://www.cottongen.org/data/download) [121]. All obtained candidate cotton *GELP* genes were further validated by analyzing the conserved Lipase_GDSL domain (IPR035669, PF00657) of the protein sequences at the website of InterPro (http://www.ebi.ac.uk/interpro/) and Pfam (http://pfam.xfam.org/). Then the cotton *GELP* family genes were renamed based on their positions on the genome and location on the chromosomes of *G. hirsutum*, *G. arboreum*, and *G. raimondii*. The visualization of chromosome location was displayed by TBtools [13, 15].

### Sequence analysis and functional annotation of cotton *GELP* genes

The detailed basic information of successfully identified cotton *GELP* genes from *G. hirsutum*, *G. arboreum*, and *G. raimondii* were extracted from the genome database and annotation files. The physiological biochemical properties of the *GELP* proteins such as molecular weight (kDa) and the theoretical isoelectric point (pI) were calculated by the ExPASy online server tool (https://www.expasy.org/). The exon–intron structure and conserved motif of the cotton *GELPs* were analyzed by Multiple Em for Motif Elicitation (MEME) online service tools (http://meme-suite.org/). The graphical visualization was displayed by TBtools. The 2-kb genomic sequence upstream of the initiation codon of the cotton *GELPs* were retrieved from the genome databases and submitted to PlantCARE website (http://bioinformatics.psb.ugent.be/webtools/plantcare/html/) [63] for investigation of the *cis*-regulatory elements. The schematic diagrams of the different *cis*-regulatory elements were drawn by the Simple BioSequence Viewer package in TBtools software. The sequence identity matrix of the GELPs at both nucleotide and amino acid levels were calculated by BioEdit (version 7.1.3.0; [40]) and displayed by MultiEperiment Viewer (MeV, version 4.9.0, TIGR, Rockville, U.S.A.) after multiple sequence alignments that were performed by MUSCLE packages of MEGA-X software (Mega, Auckland, New Zealand) with default parameters.

### Expansion and evolution analysis of *GELP* genes

The protein sequences of identified 389 *GELPs* from *G. hirsutum*, *G. arboreum*, and *G. raimondii* and 104 *Arabidopsis GELPs* were used to construct the phylogenetic tree by FastTree (version 2.1.11; http://meta.microbesonline.org/fasttree/) software [83], which was subsequently decorated through the EvolView (https://www.evolgenius.info/evolview/). The Kimura 2-parameter correction genetic distance, transition genetic distance, and transversion genetic distance between orthologous/paralogous gene pairs of the *GELP* family of cotton were evaluated by MEGA-X for base substitution saturation analysis. The synteny relationship between the *GELP* genes that evolved from the A/D subgenome was analyzed by the MCscanX program [106, 108]. All collinear gene pairs of *GELP* for WGD or segmental and tandem duplication events were extracted from the results of the synteny analysis and were visualized by Genome Gene Dotplot, Advanced Circos, and Multiple Synteny Plot packages inside the software TBtools. The values of nonsynonymous substitution rates (Ka) and synonymous substitution rates (Ks) of the evolved *GELP* orthologous or paralogous gene pairs were calculated by the software TBtools with NG methods to deduce the natural selection pressure of *GELP* genes in the process of evolution. Ka/Ks ratio < 1, = 1, and > 1 mean that the gene encounters purification selection, natural selection and positive selection, respectively. The biological meaning of Ks can be used to infer the divergence time of large-scale genome-wide and segmental duplication events that occurred within the species during evolution process, and the divergence time can be calculated [90].

### Transcriptomic expression profile analysis of *GELP* genes

The expression levels of* GELPs* in various tissues (root, stem, leaf, petal, torus, stamen, pistil, and calycle), different periods of ovule and fiber development (− 3, − 1, 0, 1, 3 dpa for ovule, and 5, 10, 20, 25 dpa for fiber), diverse stages of seed germination (seed: 0, 5, 10 h; cotyledon: 24, 48, 72, 96, 120 h; root: 24, 48, 72, 96, 120 h), and different abiotic stresses (hot, cold, drought, and salt) and biotic stress (*Verticillium dahliae* treatment for 6, 48, and 120 h), were analyzed based on the fragments per kilobase per million mapped (FPKM) value extracted from the publicly released different transcriptomes (BioProject accession: PRJNA248163, PRJNA408075) [125]. The cluster analysis of differentially expressed genes (DEGs) was completed by Short Time-series Expression Miner (STEM) software [31], and the FPKM-based heat-maps of DEGs were drawn by TBtools.

### Sample collection and real-time quantitative PCR (RT-qPCR) analysis

The upland cotton TM-1 materials of ovules and fibers at different developmental stages of −3, −1, 0, 1, and 3 dpa for ovules, and 5, 10, 15, 20, and 25 dpa for fibers were collected as the experimental samples for RT-qPCR detection analysis. The four-week-old upland cotton TM-1 leaves were sprayed with 100 μM IAA, GA, ABA, JA and 2 mM SA for 3, 6, and 9 h, respectively, with the untreated leaves as control. All the samples were immediately frozen in liquid nitrogen for further RNA extraction and cDNA synthesis. Total RNA of different materials was extracted using the RNAprep Pure Plant Plus Kit (TianGen, Beijing, China) according to the protocol guidelines. cDNA was obtained by RNA reverse transcription using FastKing gDNA Dispelling RT SuperMix kit (TianGen, Beijing, China) and was then used as templates for RT-qPCR assay with the designed gene specific primers by Primer Premier 5 software (PREMIER Biosoft, San Francisco, USA), and *GhUBQ7* (DQ116441.1) was used as internal reference for normalization (Supplementary Table S24). A total of 20 μL final volume was mixed for each reaction that was launched in Light Cycler@480 (Roche, Basel, Switzerland).

### Supplementary Information

**Additional file 1:** **Supplementary Table 1.**  The *Gossypium* genome sources used in this study. **Supplementary Table 2.** The orthologous/paralogous gene pairs of *GELPs* of *G. raimondii*, *G. arboreum*, and *G. hirsutum*. **Supplementary Table 3.** The Nucleic acid coding region and amino acid sequences of *GELP* genes of *G. hirsutum*. **Supplementary Table 4.** Analysis of the physical and chemical properties of *GELP* gene sequences in *G. raimondii*, *G. arboreum*, and *G. hirsutum*. **Supplementary Table 5.** Prediction analysis of the protein conserved structure and potential functional classification of the members of the *GELP* gene family in *G. raimondii*, *G. arboreum*, and *G. hirsutum*. **Supplementary Table 6.** The *GELP* homologous gene pairs that are collinear on each subgenome of *G. raimondii*, *G. arboreum*, and *G. hirsutum*. **Supplementary Table 7.** The rate of base substitution between sequences of *GELP* orthologous or paralogous gene pairs in *G. raimondii*, *G. arboreum*, and *G. hirsutum*. **Supplementary Table 8.** The FPKM value of the expression level of *GELP* genes specifically expressed in different tissues in *G. raimondii*, *G. arboreum*, and *G. hirsutum*. **Supplementary Table 9.** The FPKM value of the expression of *GELP* genes during the fiber initiation and fiber growth in *G. hirsutum*. **Supplementary Table 10.** The FPKM value of *GELP* genes expression in different tissues during seed germination in *G. hirsutum*. **Supplementary Table 11.** The FPKM value of expression of *GELP* genes in response to abiotic stress in *G. hirsutum*. **Supplementary Table 12.** The FPKM value of expression of *GELP* genes in response to stress from *Verticillium dahliae* in *G. hirsutum*. **Supplementary Table 13.** Up-regulated and down-regulated expressed *GELP* genes at the initial stage of fiber development. **Supplementary Table 14.** Up-regulated and down-regulated expressed *GELP* genes during fiber development. **Supplementary Table 15.** Up-regulated and down-regulated expressed *GELP* genes during the initial stage of seed germination. **Supplementary Table 16.** Up-regulated and down-regulated expressed *GELP* genes in root tissues after seed germination. **Supplementary Table 17.** Up-regulated and down-regulated expressed *GELP* genes in cotyledons after seed germination. **Supplementary Table 18.** Up-regulated and down-regulated expressed *GELP* genes in response to 20% PEG stress. **Supplementary Table 19.** Up-regulated and down-regulated expressed *GELP* genes in response to 4℃ low temperature stress. **Supplementary Table 20.** Up-regulated and down-regulated expressed *GELP* genes in response to 38℃ high temperature stress. **Supplementary Table 21.** Up-regulated and down-regulated expressed *GELP* genes after 400 mM NaCl stress treatment. **Supplementary Table 22.** Up-regulated and down-regulated expressed *GELP* genes in response to *Verticillium dahliae*. **Supplementary Table 23.** Statistics of the number of hormone-responsive *cis*-acting elements locating on the promoters of *GELP* family members in *G. raimondii*, *G. arboreum*, and *G. hirsutum*. **Supplementary Table 24.** The RT-qPCR primers used to detect the relative expression of *GhGELPs* during cotton fiber development and in response to plant hormone treatment.

**Additional file 2:** **Supplementary Figure S1. **Gene structure and conserved motif analysis of *GELPs* in *G. arboreum, G. raimondii and G. hirsutum*. A total of 389 GELPs from *Gossypium hirsutum*, *G. arboreum* , and *G. raimondii* are subjected for clustering with different groups of Group I—Group IIId. Exons and introns were indicated by black lines and blue boxes, respectively, with genomic lengths showed at the centre. Ten conserved motifs distributing in GELPs are displayed with different colored boxes, showing the amino acid length at the centre. The specific amino acid sequences of the ten conserved motifs are showed by different colored letters, with the height of each letter representing the frequency of amino acids at that position. **Supplementary Figure S2. **Expression profiles of *GELP* genes in different tissues of *G. hirsutum*. A total of 193 *GhGELPs *from upland cotton *G. hirsutum* are subjected for clustering with different clusters indicated by different colored lines. The fragments per kilobase of exon model per million (FPKM) value obtained from the publicly released transcriptome data of different cotton tissues was collected for expression profile analysis. The solid different colored dot size denotes the different expression levels with big and red dots for high expression levels and blue and small dots for low expression levels. The visualization of the FPKM-based transcriptome data was generated by TBtools software. **Supplementary Figure S3.** Expression profiles of *GELP* genes during fiber growth and development in *G. hirsutum*. The 193 *GhGELPs* from upland cotton *G. hirsutum* are subjected for clustering with different clusters indicated by different colored lines. The FPKM value obtained from the publicly released transcriptome data of different periods of cotton ovules and fibers (−3, −1, 0, 1, 3 dpa for ovules, and 5, 10, 20, 25 dpa for fibers) was collected for expression profile analysis. The solid different colored dot size represents the different expression levels with big and red dots for high expression levels and blue and small dots for low expression levels. The visualization of the FPKM-based transcriptome data was generated by TBtools software. **Supplementary Figure S4. **Expression profiles of *GELP* genes in different tissues during seed germination in *G. hirsutum*. The 193 *GhGELPs* from upland cotton *G. hirsutum* are subjected for clustering with different clusters indicated by different colored lines. The FPKM value obtained from the publicly released transcriptome data of different cotton tissues of seed (0 h, and post germination of 5 h and 10 h), cotyledon (24, 48, 72, 96, and 120 h), and root (48, 72, 96, and 120 h) was collected for expression profile analysis. The solid different colored dot size represents the different expression levels with big and red dots for high expression levels and blue and small dots for low expression levels. The visualization of the FPKM-based transcriptome data was generated by TBtools software. **Supplementary Figure S5. **Expression profile of *GELP* genes in response to abiotic stress in *G. hirsutum*.The 193 *GhGELPs* from upland cotton *G. hirsutum* are subjected for clustering with different clusters indicated by different colored lines. The FPKM value obtained from the publicly released transcriptome data of different cotton tissues treated by diverse abiotic stresses of cold, hot, salt, and polyethylene glycol (PEG) for 0, 1, 3, 6, and 12 h was collected for expression profile analysis. The solid different colored dot size denotes the different expression levels with big and red dots for high expression levels and blue and small dots for low expression levels. The visualization of the FPKM-based transcriptome data was generated by TBtools software. **Supplementary Figure S6. **Expression profile of *GELP* genes in response to *Verticillium dahliae* treatment in *G. hirsutum. *The 193 *GhGELPs* from upland cotton *G. hirsutum* are subjected for clustering with different clusters indicated by different colored lines. The FPKM value obtained from the publicly released transcriptome data of cotton roots incubated with *V. dahliae* for 0, 6, 48, and 120 h was collected for expression profile analysis. The solid different colored dot size denotes the different expression levels with big and red dots for high expression levels and blue and small dots for low expression levels. The visualization of the FPKM-based transcriptome data was generated by TBtools software. **Supplementary Figure S7. **Expression analysis of co-expressed *GhGELPs* of *G. hirsutum *in different processes of growth, development, and stress response. Expression profile features of the co-expressed* GhGELPs* in different processes of fiber initiation and growth (a), seed germination (b), diverse abiotic stress (c), and biotic stress (*Verticillium dahliae*) were analyzed. The FPKM value obtained from the publicly released transcriptome data was collected for expression profile analysis. The diverse colors denote the different expression levels with red and orange for high expression levels and blue and green for low expression levels. The heat-maps were visualized by the FPKM-based transcriptome data using TBtools software. **Supplementary Figure S8. **Putative*cis*-elements of the promoters of *GELP* genes from *G. arboreum,G. raimondii*, and *G. hirsutum*. The 2000-bp promoter sequences of the 389 *GELPs* from *G. hirsutum*, *G. arboreum* , and *G. raimondii* are subjected for *cis*-element analysis by PlantCARE software. The promoter sequences were indicated by solid lines, the diverse *cis*-elements distributing on the promoters were showed by different colored boxes, with the promoter sequence length exhibited at the centre. The visualized figure was generated by TBtools software.

## Data Availability

All data generated or analyzed during this study are included in this published article.
