# Supplementary material for: Identification, evolution, and expression of GDSL-type Esterase/Lipase (GELP) gene family in three cotton species: a bioinformatic analysis
Source: BMC Genomics. 2023 Dec 21;24:795. doi: 10.1186/s12864-023-09717-3 (PMC10734139; doi:10.1186/s12864-023-09717-3)
Supplement: Supplementary file 2 — Additional file 2: Supplementary Figure S1. Gene structure and conserved motif analysis of GELPs in G. arboreum, G. raimondii and G. hirsutum. A total of 389 GELPs from Gossypium hirsutum, G. arboreum , and G. raimondii are subjected for clustering with different groups of Group I—Group IIId. Exons and introns were indicated by black lines and blue boxes, respectively, with genomic lengths showed at the centre. Ten conserved motifs distributing in GELPs are displayed with different colored boxes, showing the amino acid length at the centre. The specific amino acid sequences of the ten conserved motifs are showed by different colored letters, with the height of each letter representing the frequency of amino acids at that position. Supplementary Figure S2. Expression profiles of GELP genes in different tissues of G. hirsutum. A total of 193 GhGELPs from upland cotton G. hirsutum are subjected for clustering with different clusters indicated by different colored lines. The fragments per kilobase of exon model per million (FPKM) value obtained from the publicly released transcriptome data of different cotton tissues was collected for expression profile analysis. The solid different colored dot size denotes the different expression levels with big and red dots for high expression levels and blue and small dots for low expression levels. The visualization of the FPKM-based transcriptome data was generated by TBtools software. Supplementary Figure S3. Expression profiles of GELP genes during fiber growth and development in G. hirsutum. The 193 GhGELPs from upland cotton G. hirsutum are subjected for clustering with different clusters indicated by different colored lines. The FPKM value obtained from the publicly released transcriptome data of different periods of cotton ovules and fibers (−3, −1, 0, 1, 3 dpa for ovules, and 5, 10, 20, 25 dpa for fibers) was collected for expression profile analysis. The solid different colored dot size represents the different expression [file 12864_2023_9717_MOESM2_ESM.docx]

| 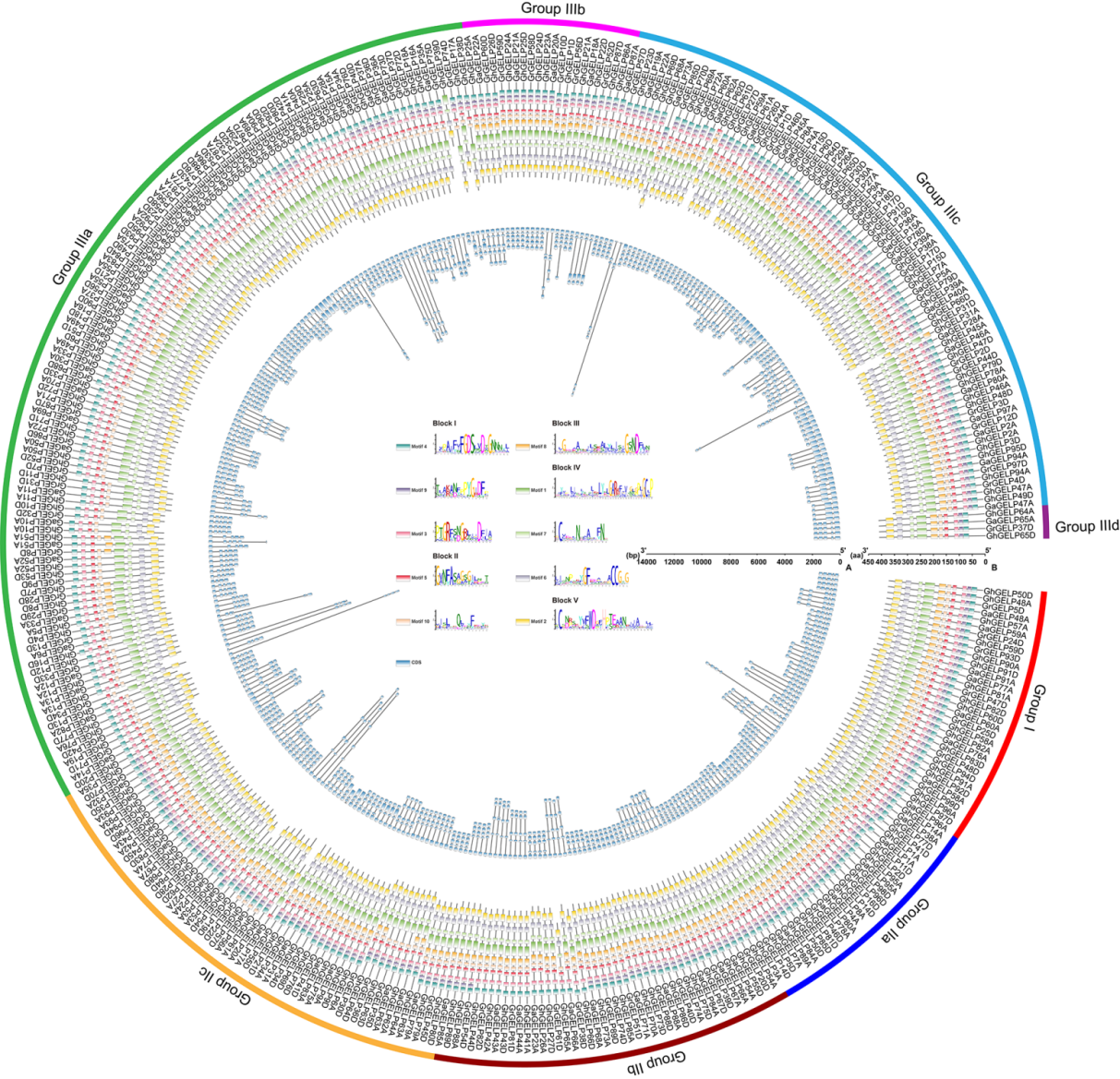 |
| --- |
| **Supplementary Figure S1. Gene structure and conserved motif analysis of *GELPs* in *G.*** *arboreum****, G. raimondii and G. hirsutum*.** A total of 389 GELPs from *Gossypium hirsutum*, *G. arboreum* , and *G. raimondii* are subjected for clustering with different groups of Group Ⅰ—Group Ⅲd. Exons and introns were indicated by black lines and blue boxes, respectively, with genomic lengths showed at the centre. Ten conserved motifs distributing in GELPs are displayed with different colored boxes, showing the amino acid length at the centre. The specific amino acid sequences of the ten conserved motifs are showed by different colored letters, with the height of each letter representing the frequency of amino acids at that position. |

| 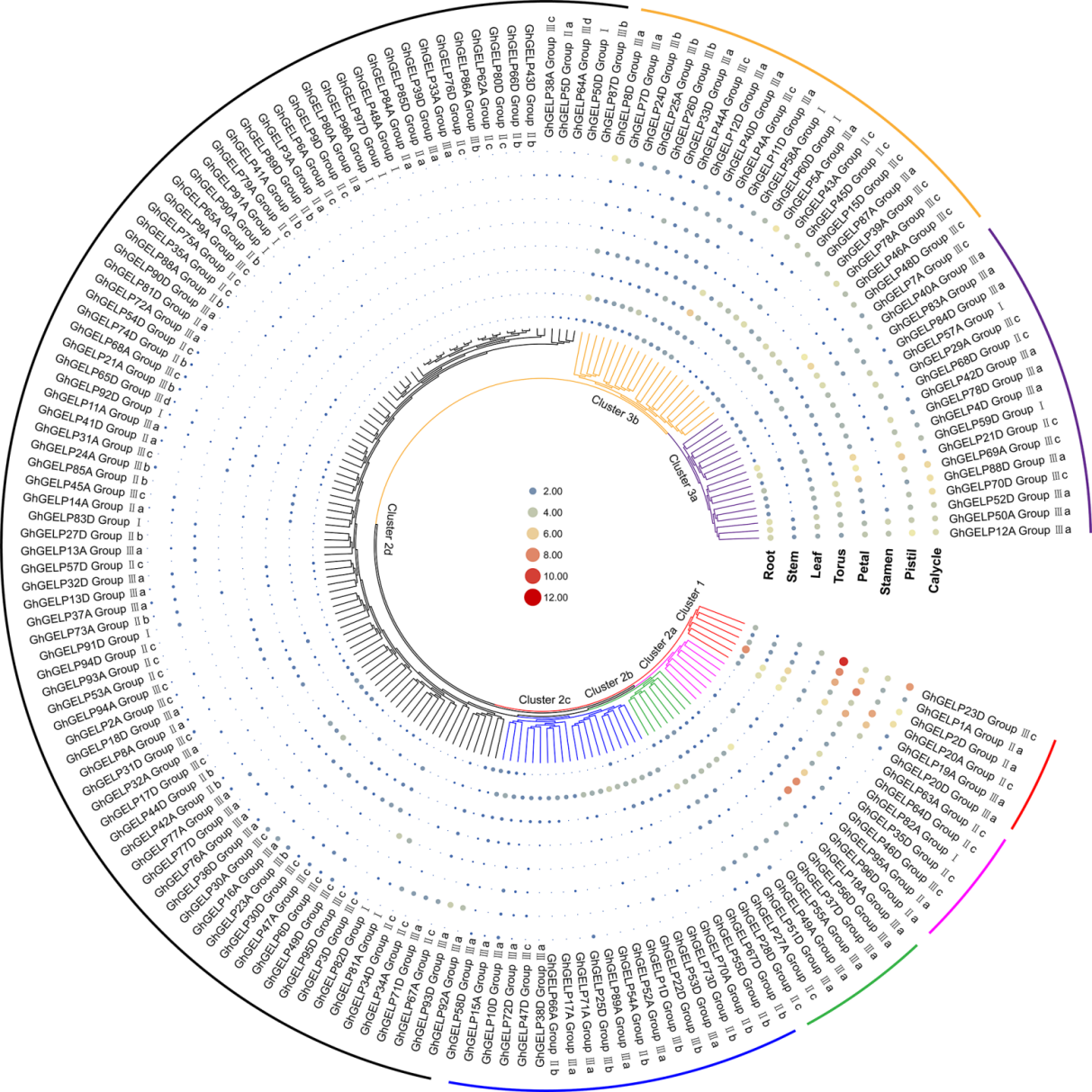 |
| --- |
| **Supplementary Figure S2. Expression profiles of *GELP* genes in different tissues of *G. hirsutum*.** A total of 193 *GhGELPs* from upland cotton *G. hirsutum* are subjected for clustering with different clusters indicated by different colored lines. The fragments per kilobase of exon model per million (FPKM) value obtained from the publicly released transcriptome data of different cotton tissues was collected for expression profile analysis. The solid different colored dot size denotes the different expression levels with big and red dots for high expression levels and blue and small dots for low expression levels. The visualization of the FPKM-based transcriptome data was generated by TBtools software. |

| 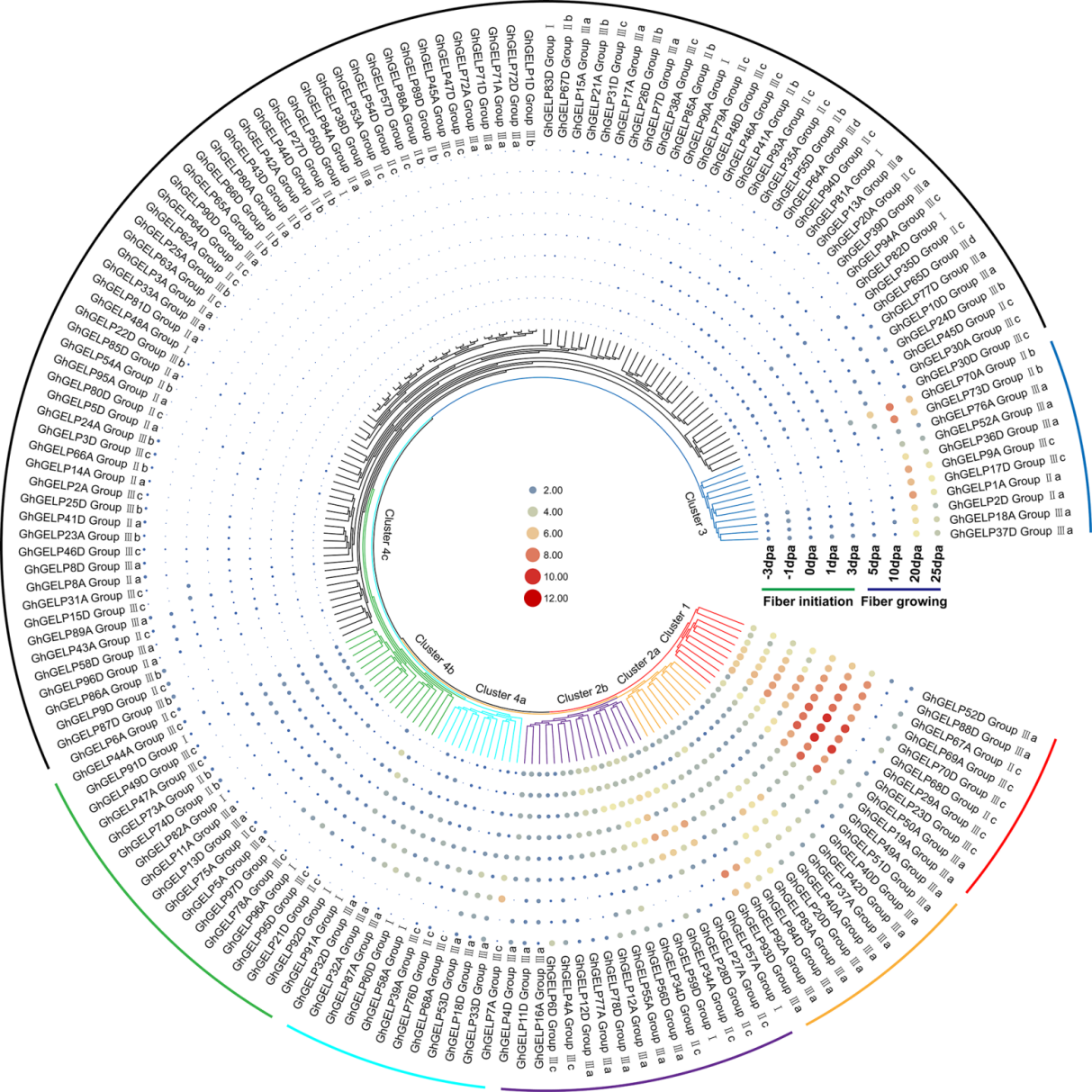 |
| --- |
| **Supplementary Figure S3. Expression profiles of *GELP* genes during fiber growth and development in *G. hirsutum*.** The 193 *GhGELPs* from upland cotton *G. hirsutum* are subjected for clustering with different clusters indicated by different colored lines. The FPKM value obtained from the publicly released transcriptome data of different periods of cotton ovules and fibers (-3, -1, 0, 1, 3 dpa for ovules, and 5, 10, 20, 25 dpa for fibers) was collected for expression profile analysis. The solid different colored dot size represents the different expression levels with big and red dots for high expression levels and blue and small dots for low expression levels. The visualization of the FPKM-based transcriptome data was generated by TBtools software. |

| 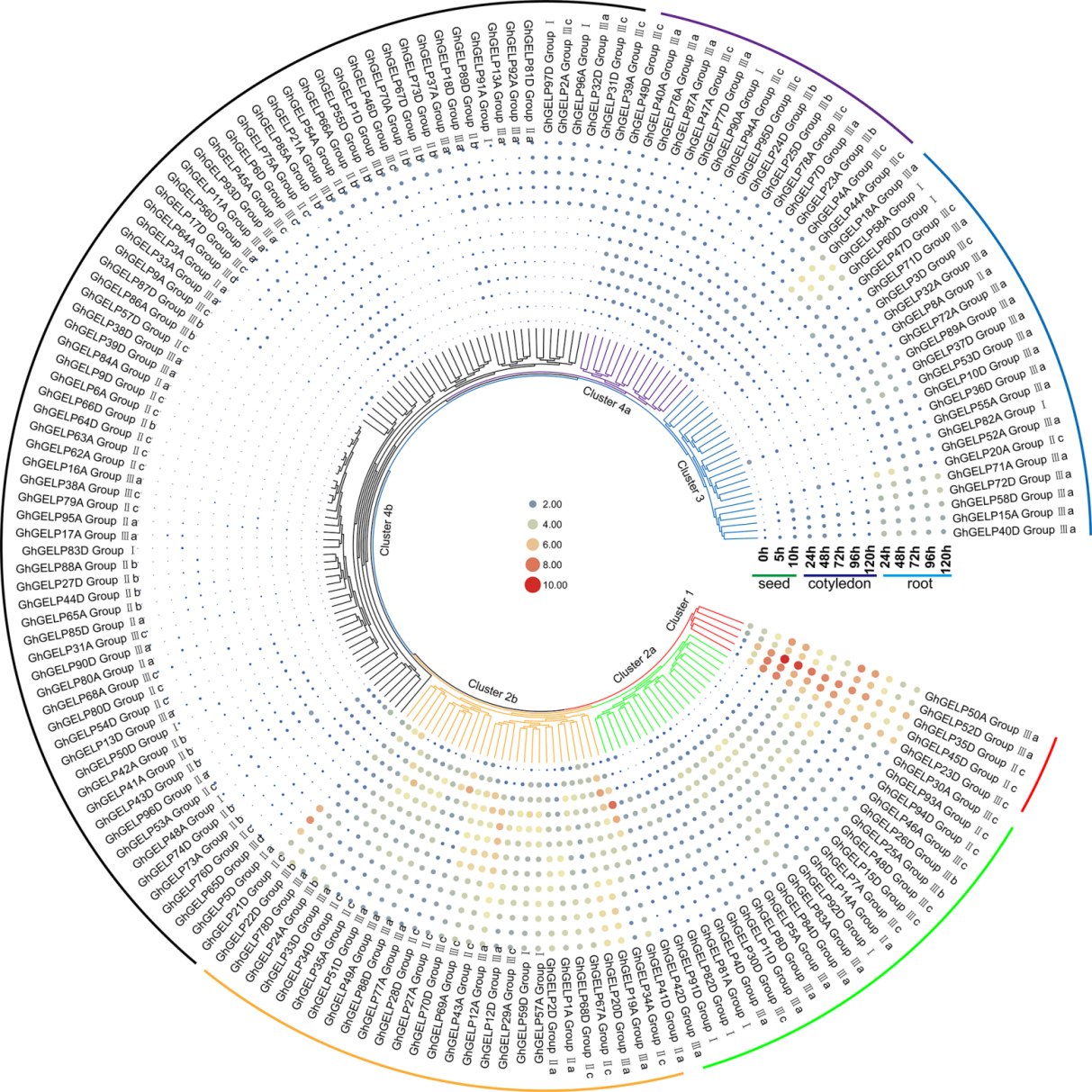 |
| --- |
| **Supplementary Figure S4. Expression profiles of *GELP* genes in different tissues during seed germination in *G. hirsutum*.** The 193 *GhGELPs* from upland cotton *G. hirsutum* are subjected for clustering with different clusters indicated by different colored lines. The FPKM value obtained from the publicly released transcriptome data of different cotton tissues of seed (0 h, and post germination of 5 h and 10 h), cotyledon (24, 48, 72, 96, and 120 h), and root (48, 72, 96, and 120 h) was collected for expression profile analysis. The solid different colored dot size represents the different expression levels with big and red dots for high expression levels and blue and small dots for low expression levels. The visualization of the FPKM-based transcriptome data was generated by TBtools software. |

| 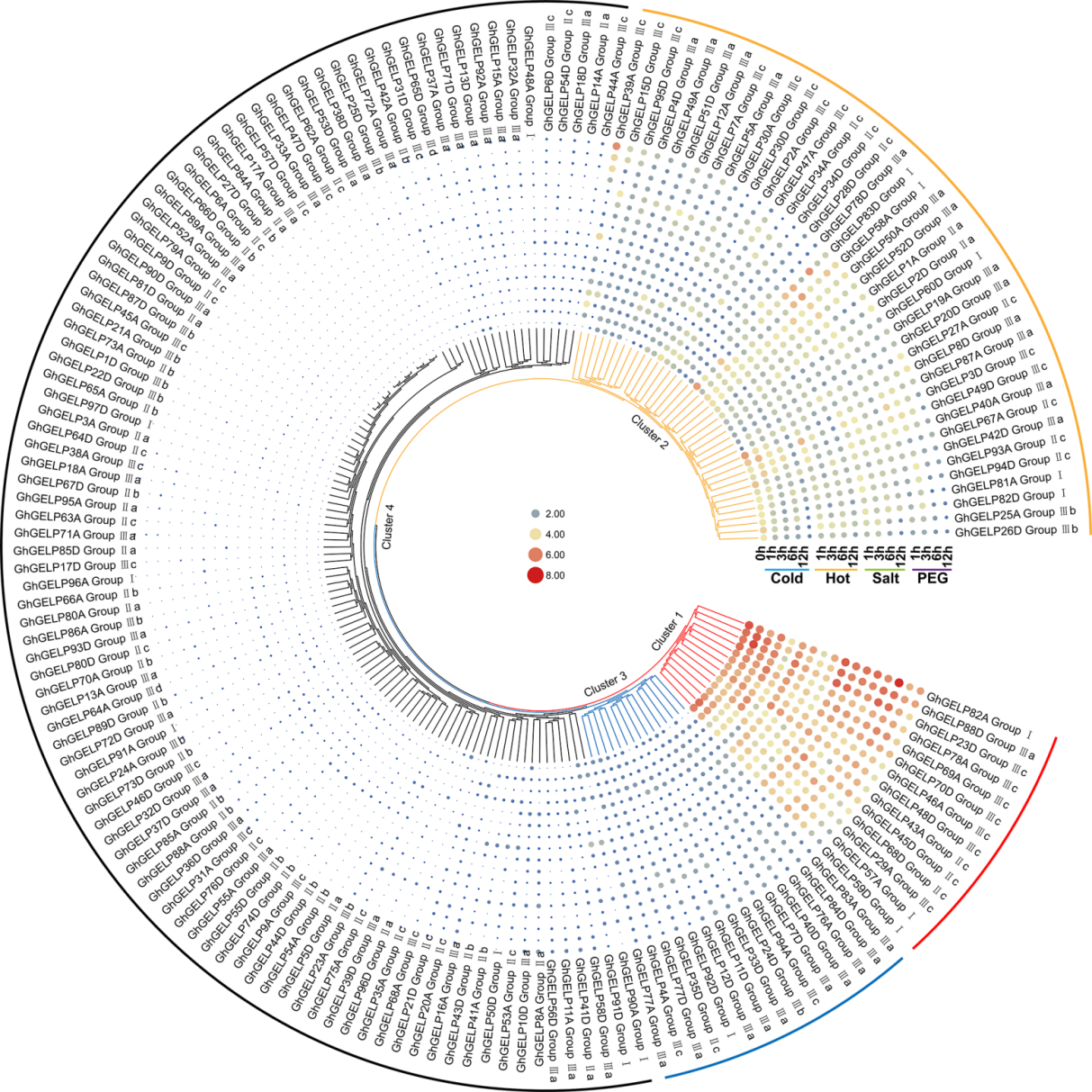 |
| --- |
| **Supplementary Figure S5. Expression profile of *GELP* genes in response to abiotic stress in *G. hirsutum*.** The 193 *GhGELPs* from upland cotton *G. hirsutum* are subjected for clustering with different clusters indicated by different colored lines. The FPKM value obtained from the publicly released transcriptome data of different cotton tissues treated by diverse abiotic stresses of cold, hot, salt, and polyethylene glycol (PEG) for 0, 1, 3, 6, and 12 h was collected for expression profile analysis. The solid different colored dot size denotes the different expression levels with big and red dots for high expression levels and blue and small dots for low expression levels. The visualization of the FPKM-based transcriptome data was generated by TBtools software. |

| 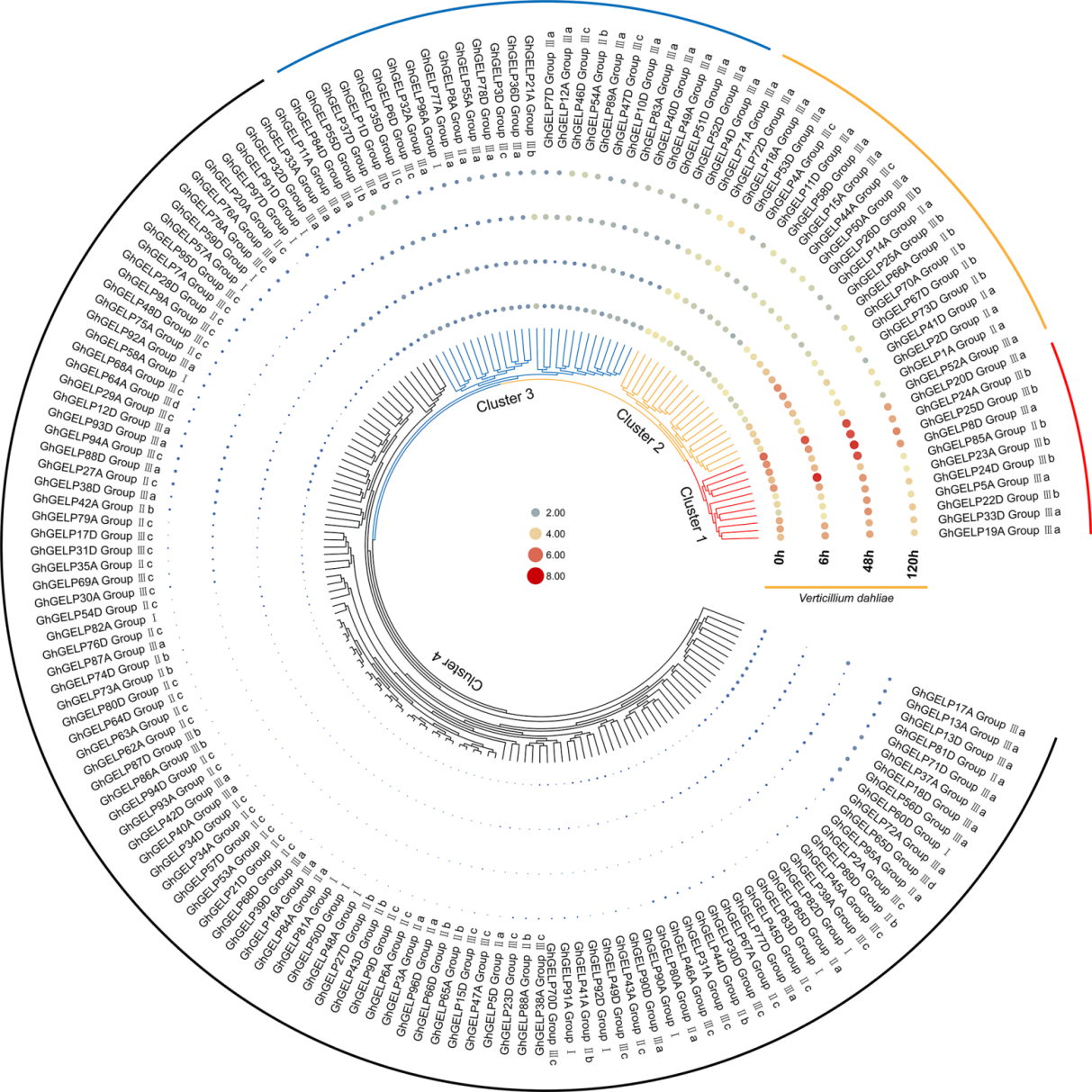 |
| --- |
| **Supplementary Figure S6. Expression profile of *GELP* genes in response to *Verticillium dahliae* treatment in *G. hirsutum.*** The 193 *GhGELPs* from upland cotton *G. hirsutum* are subjected for clustering with different clusters indicated by different colored lines. The FPKM value obtained from the publicly released transcriptome data of cotton roots incubated with *V. dahliae* for 0, 6, 48, and 120 h was collected for expression profile analysis. The solid different colored dot size denotes the different expression levels with big and red dots for high expression levels and blue and small dots for low expression levels. The visualization of the FPKM-based transcriptome data was generated by TBtools software. |

| 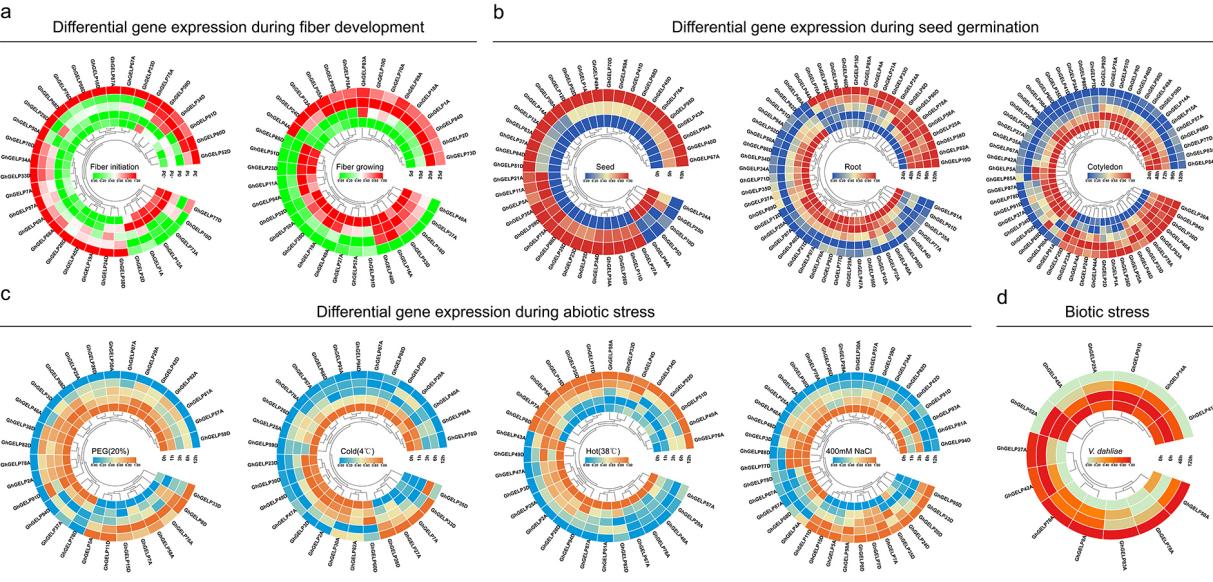 |
| --- |
| **Supplementary Figure S7. Expression analysis of co-expressed *GhGELPs* of *G. hirsutum* in different processes of growth, development, and stress response.** Expression profile features of the co-expressed *GhGELPs* in different processes of fiber initiation and growth (a), seed germination (b), diverse abiotic stress (c), and biotic stress (*Verticillium dahliae*) were analyzed. The FPKM value obtained from the publicly released transcriptome data was collected for expression profile analysis. The diverse colors denote the different expression levels with red and orange for high expression levels and blue and green for low expression levels. The heat-maps were visualized by the FPKM-based transcriptome data using TBtools software. |

| 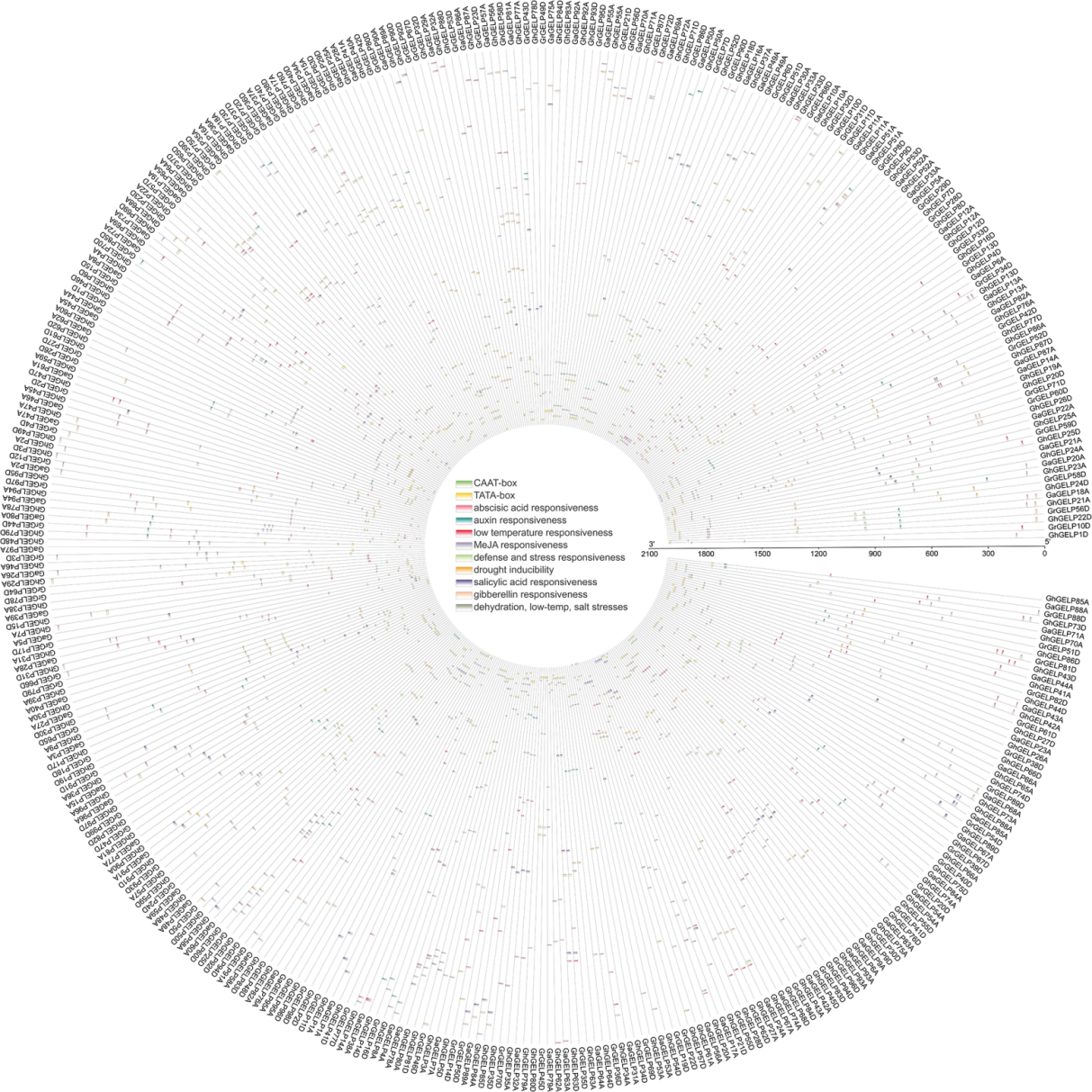 |
| --- |
| **Supplementary Figure S8. Putative *cis*-elements of the promoters of *GELP* genes from *G. arboreum,* *G. raimondii*, and *G. hirsutum*.** The 2000-bp promoter sequences of the 389 *GELPs* from *G. hirsutum*, *G. arboreum* , and *G. raimondii* are subjected for *cis*-element analysis by PlantCARE software. The promoter sequences were indicated by solid lines, the diverse *cis*-elements distributing on the promoters were showed by different colored boxes, with the promoter sequence length exhibited at the centre. The visualized figure was generated by TBtools software. |
